# A Transcriptomic Immunologic Signature Predicts Favorable Outcome in Neoadjuvant Chemotherapy Treated Triple Negative Breast Tumors

**DOI:** 10.3389/fimmu.2019.02802

**Published:** 2019-12-18

**Authors:** Javier Pérez-Pena, Janos Tibor Fekete, Raquel Páez, Mariona Baliu-Piqué, José Ángel García-Saenz, Vanesa García-Barberán, Aránzazu Manzano, Pedro Pérez-Segura, Azucena Esparis-Ogando, Atanasio Pandiella, Balázs Gyorffy, Alberto Ocana

**Affiliations:** ^1^Experimental Therapeutics Unit, Medical Oncology Department, Hospital Clínico San Carlos (HCSC), Instituto de Investigación Sanitaria (IdISSC) and CIBERONC, Madrid, Spain; ^2^Department of Bioinformatics, Semmelweis University, Budapest, Hungary; ^3^Department of Paediatrics, Semmelweis University, Budapest, Hungary; ^4^TTK Lendület Cancer Biomarker Research Group, Institute of Enzymology, Hungarian Academy of Sciences (MTA), Budapest, Hungary; ^5^Translational Research Unit, Translational Oncology Laboratory, Albacete University Hospital, Albacete, Spain; ^6^Centro Regional de Investigaciones Biomedicas, Castilla-La Mancha University (CRIB-UCLM), Albacete, Spain; ^7^Instituto de Biología Molecular y Celular del Cáncer and CIBERONC, CSIC-Universidad de Salamanca, Salamanca, Spain

**Keywords:** immunotherapy, triple negative breast cancer, chemotherapy treated patients, transcriptomic signature, outcome

## Abstract

Limited therapeutic options exist for the treatment of patients with triple negative breast cancer (TNBC). Neoadjuvant chemotherapy is currently the standard of care treatment in the early stages of the disease, although reliable biomarkers of response have been scarcely described. In our study we explored whether immunologic signatures associated with inflamed tumors or hot tumors could predict the outcome to neoadjuvant chemotherapy. Publicly available transcriptomic data of more than 2,000 patients were evaluated. ROC plots were generated to assess the response to therapy. Cox proportional hazards regression was computed. Kaplan-Meier plots were drawn to visualize the survival differences. Higher expression of IDO1, CXCL9, CXCL10, HLA-DRA, HLA-E, STAT1, and GZMB were associated with a higher proportion without relapse in the first 5 y after chemotherapy in TNBC. The expression of these genes was associated with a high presence of CD8 T cells in responder patients using the EPIC bioinformatic tool. The strongest effect was observed for STAT1 (*p* = 1.8e-05 and AUC 0.69, *p* = 2.7e-06). The best gene-set signature to predict favorable RFS was the combination of IDO1, LAG3, STAT1, and GZMB (HR = 0.28, CI = 0.17–0.46, *p* = 9.8 E-08, FDR = 1%). However, no influence on pathological complete response (pCR) was observed. Similarly, no benefit was identified in any other tumor subtype: HER2 or estrogen receptor positive. In conclusion, we describe a set of immunologic genes that predict the outcome to neoadjuvant chemotherapy in TNBC, but not pCR, suggesting that this non-time to event endpoint is not a good surrogate marker to detect the long term outcome for immune activated tumors.

## Introduction

Breast cancer is a heterogeneous disease classified in several subgroups based on molecular and genomic profiles (1). The triple negative breast cancer (TNBC) subtype is represented by tumors that lack the presence of hormone receptors and HER2 overexpression on the cell membrane (2). This group constitutes the majority of the basal-like subtype described by gene expression analyses (2). In contrast to other breast cancer subgroups, where targeted therapies have been developed including several forms of anti-estrogen therapies in hormone receptor positive tumors, or anti-HER2 therapies -nude or loaded antibodies, or small tyrosine kinase inhibitors- in HER2 positive breast cancers (3); the triple negative subtype lacks specific forms of systemic treatment (2).

Recently, atezolizumab, a specific immunotherapy agent that targets an immunologic checkpoint receptor with an antibody against its ligand, PD-L1, has shown clinical activity leading to its regulatory approval (4). Although some studies have reported the association between the expression of PD-L1 or tumor mutational burden with patient benefit, this association is only observed in some tumors (5, 6). In this context, the lack of biomarkers to select those patients who are likely to respond, still limits their efficacy.

In any case, what is recognized is that those tumors that have a pre-existing stage of immunologic T-cell activation,—what has been termed “hot tumors”—would respond better to check point inhibitors (5, 7). Preceding this, a key and initial process within the immune activation cascade is the presentation of tumoral antigens to effector T cells (8–10).

It has been suggested that chemotherapy can augment the efficacy of immunotherapies by simultaneously accumulating neoantigens, thereby augmenting the activation of CD8 T cells; and also by inducing genomic instability that increases the mutational load (7). If this is the case, the mere use of chemotherapy might stimulate the immune response, particularly in those tumors with a pre-existing stage of immunologic activation.

To test this hypothesis, we explored whether described immunologic signatures are able to predict the response to chemotherapy. We used a population of TNBC patients treated with neoadjuvant chemotherapy and confirmed the results in another cohort of early stage breast cancer patients. Our transcriptomic signature provides evidence that hot TNBC tumors respond better to chemotherapy evaluating relapse free survival (RFS), opening the possibility to further explore this set of genes in the clinical setting.

## Materials and Methods

### Prediction of Neoadjuvant Therapies

We have set up a database of transcriptomic datasets with available response and treatment data used to evaluate the relationship between the expression of those genes of interest and patients' response to different treatments, including chemotherapy, anti HER2 therapies, and endocrine therapies. In this, a PubMed search lead us to the identification of 2,108 breast cancer samples who received chemotherapy treatment in the neoadjuvant setting and for whom the gene expression was measured using Affymetrix HGU133A and HGU133A plus 2.0 microarrays. We specifically included the following datasets in the analysis: E-MTAB-365, GSE45255, GSE19615, GSE2603, GSE21653, GSE31519, and GSE37946. We used those in which different treatments were included. We added this data to the ROC Plotter Online Tool (http://www.rocplot.org). Pathological complete response was defined as a complete response vs. any residual disease after completion of therapy. The two cohorts were compared using a ROC analysis by a Mann-Whitney test. In addition to the Area Under the Curve (AUC) and *p*-values, the strongest cutoff, the False Positive Rate (FPR), and the True Positive Rate (TPR) were calculated for each gene. Finally, FDR was computed and only results with an FDR < 10% were accepted as significant. Data about patients' characteristics is provided in Supplementary Table 1. First, each gene was analyzed using a gene expression as a continuous variable. Then, for the Kaplan-Meier plots, the calculation of the minimal *p*-value cut-off was performed by first determining each cut-off value between the lower and upper quartiles of the expression. Then, these cut-off values were used to divide the patients into two cohorts, and these cohorts were compared in the Cox regression analysis. The *p*-values for each analysis were noted and the minimal *p*-value was used when drawing the Kaplan-Meier plots. FDR was computed using all the derived cut-off values, and only results with an FDR below 10% were accepted as significant. FDR was calculated using all the available p values by employing the brainwaver library (https://cran.r-project.org/web/packages/brainwaver/brainwaver.pdf) in the R statistical environment.

### Outcome Analyses

The KM Plotter Online Tool (http://www.kmplot.com) was used to evaluate the relationship between the presence of different genes and the patient clinical outcomes in different breast cancer subtypes (11). This publicly available online tool allowed us to investigate relapse-free survival (RFS) in TNBC. This subtype was defined as ER-/PR-/HER2-. The best performing threshold between low and high expression was used as a cutoff. We also used a cohort of adjuvant TNBC patients treated only with chemotherapy to confirm the results.

### Statistical Analyses

Kaplan-Meier plots were drawn to visualize the survival differences. Cox proportional hazards regression was computed to explore the association between the gene expression and outcomes. Multiple genes were combined into a signature using their mean expression. Statistical significance was defined as *p* < 0.05.

## Results

### Selection of Immunologic Signatures and Response to Neoadjuvant Chemotherapy in Triple Negative Breast Cancer

**Figure 1:**
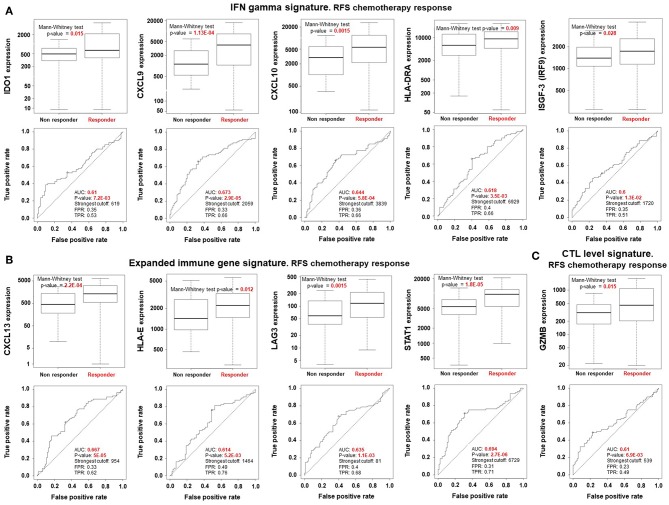
Results of immune related genes that predict better outcome in chemotherapy treated TNBC patients. Box-plots comparing responders (higher proportion without relapse in the first 5 years after) vs. non-responders using a Mann-Whitney test and the Area Under the Curve (AUC) [*p*-values, the strongest cutoff, the False Positive Rate (FPR), and the True Positive Rate (TPR)], were calculated for each gene for: **(A)** INF gamma signature, **(B)** expanded immune gene signature, and **(C)** CTL level signature. All the *p*-values are significant at FDR < 10%.

We first selected reported signatures that identify immune inflamed tumors that are associated with a favorable response to immunomodulators (12, 13). For this purpose we included the IFN gamma signature, the expanded immune signature, the cytotoxic T lymphocyte level (CTL) signature, and the expression of HLA A and HLA B [Table 1; (10, 11)]. The individual association of gene expression with RFS at 5 years was evaluated using ROC analysis and computing the AUC value. A full description of this application is provided in the Material and Methods section. Figure 1 shows for TNBC patients treated with chemotherapy whose individual gene's expression was significantly associated with a higher proportion without relapse and their respective ROC curves. These include IDO1, CXCL9, CXCL10, HLA-DRA, and ISGF-3 from the IFN gamma signature (Figure 1A); CXCL13, HLA-E, LAG3, and STAT1 from the expanded gene signature (Figure 1B); and GZMB from the CTL-level signature (Figure 1C). The highest effect was observed for STAT1 (response: *p*-value of 1.8e-05 and an AUC 0.69, *p* = 2.7e-06). When controlling for false positives, all the *p*-values were significant at FDR < 10%. Our data suggest that these identify genes in inflamed tumors were linked with a higher proportion without relapse in the first 5 years following chemotherapy. On the other hand, we evaluated the predictive capacity of these genes in relation to pathological complete response (pCR). We observed that their presence did not identify those tumors more linked with response, suggesting that pCR is not the best endpoint when evaluating immunologically hot tumors (Supplementary Figure 1).

**Table 1:** Table of genes included in the four immunological signatures used for our analyses.

**Signature**	**Genes**
HLA	*HLA-A, HLA-B*
IFN gamma signature	*IDO1, CXCL10, CXCL9, HLA-DRA, ISGF-3, IFNG*
Expanded immune gene signature	*CD30, IDO1, CIITA, CD3E, CCL5, GZMK, CD2, HLA-DRA, CXCL13, IL2RG, NKG7, HLA-E, CXCR6, LAG3, TAGAP, CXCL10, STAT1, GZMB*
Cytotoxic T lymphocyte (CTL) level signature	*CD8A, CD8B, GZMA, GZMB, PRF1*

We have also estimated, using the EPIC tool (14), the composition of the tumor microenvironment. Evaluating our own gene signature: IDO1, CXCL9, CXCL10, CXCL13, HLA-DRA, HLA-E, IRF9, LAG3, STAT1, and GZMB, there is a clear statistical significance difference (*p* = 0.0008) in the presence of CD8 T cells between relapsed and non-relapsed TNBC patients in the first 5 years, being higher on those who did not relapse in the first 5 years (Supplementary Figure 2).

In an additional effort to identify genes that could be associated with clinical outcome we included known targets and intracellular mediators of the immune response including transcription factors, as shown in the Supplementary Table 2. Of note four known targets for which therapies are currently in clinical development showed a positive association with RFS at 5 years, including ICOS, TIGIT, CTLA-4, and CD274 (Supplementary Table 2). However, as these are well known targets in immuno-oncology we decided not to include these genes in the gene expression signature.

### Confirmation of Gene Expression With Favorable Outcome

**Figure 2:**
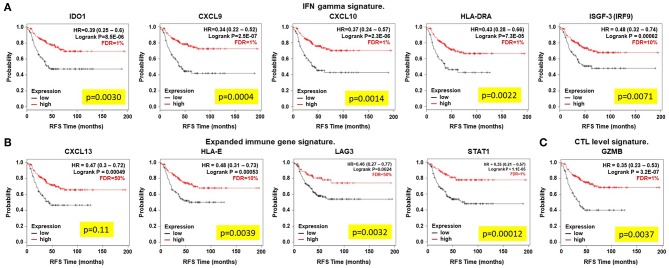
Overexpression of immune related genes that predict outcome (RFS) in early stage chemotherapy treated TNBC patients. Kaplan-Meier plots show survival differences. **(A)** INF gamma signature, **(B)** expanded immune gene signature, and **(C)** CTL level signature. Correlation to survival was analyzed when using the gene expression as a continuous variable in the Cox regression (yellow box).

We next confirmed that the expression of the identified genes predicted favorable outcome in another dataset of early stage TNBC tumors, assuming that most of early stage patients with this subtype receive chemotherapy. To do so, we used patient data included in the KM Plotter online tool as described in material and methods and published elsewhere (11). Higher expression of each of these genes predicted favorable outcome (RFS), IDO (HR = 0.39, CI = 0.25–0.6, log rank *p* = 8.5E-06), CXCL9 (HR = 0.34, CI = 0.22−0.52, log rank *p* = 2.5E-07), CXCL10 (HR = 0.37, CI = 0.24–0.57, log rank *p* = 2.3E-06), HLA-DRA (HR = 0.43, CI = 0.28–0.66, log rank *p* = 7.3E-05), ISGF-3 (HR = 0.48, CI = 0.32–0.74, log rank *p* = 0.00062), CXCL-13 (HR = 0.47, CI = 0.3–0.72, *p* = 0.00049), HLA-E (HR = 0.48, CI = 0.31–0.73, *p* = 0.00053), LAG3 (HR = 0.46, CI = 0.27–0.77, log rank *p* = 0.0024), STAT1 (HR = 0.35, CI = 0.21–0.57, log rank *p* = 1.1E-05), and GZMB (HR = 0.35, CI = 0.23–0.53, log rank *p* = 3.2E0.7; Figures 2A–C). Of note, four of these genes showed an FDR equal or higher than 10%, *CXCL13 and LAG3 (FDR* > *10%), and HLA-E and ISGF-3* (*FDR* = *10%*), compromising the significance of these results in the confirmatory cohort. Finally, we included a cohort of patients with TNBC only treated with chemotherapy, assuming that the number of patients will be reduced. As shown in Supplementary Figure 3 the results are confirmed although the FDR increases in some genes due to the reduced number of patients (114) included in this cohort.

### Identification of Best Gene-Set Combinations to Predict Outcome

**Figure 3:**
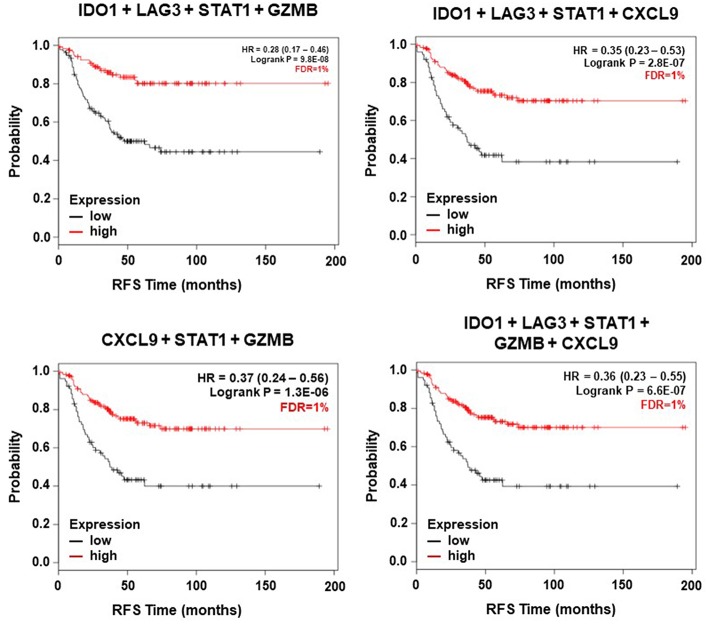
Association of immune related signatures (including the genes with the highest AUC) and relapse free survival for early stage TNBC.

Once we identified a set of genes that individually predict relapse and favorable outcome in TNBC, we aimed to uncover which combination of genes could increase the predictive power conserving a low FDR. Table 2 shows all the potential combinations for each set of genes. The best signatures were those that included combinations of: IDO1, LAG3, STAT1, CXCL9, and GZMB. The best combination included IDO1, CXCL9, STAT1, LAG3, GZMB, AUC 0.699, *p* = 1.10-E05. The same combination but excluding LAG3 showed a similar result. The combination of IDO1, LAG3, STAT1, and GZMB predicted RFS with an AUC 0.697, *p* = 1.40-E05. All other combinations are listed in Table 2. In line with this, we aimed to confirm these signatures in early stage TNBC patients. As displayed in Figure 3, the combination of IDO1, LAG3, STAT1, and GZMB showed the best favorable prognosis (HR = 0.28, CI = 0.17–0.46, *p* = 9.8 E-08 and FRD = 1%). We performed Cox multivariate regression analysis including nodal status, grade, age, and the combined signature. In this, the signature of IDO1+LAG3+STAT1+GZMB reached the highest significance (*p* = 1.9E-04), nodal status was also significant (*p* = 6.1E-04), while grade and age were not significant. Other combinations showed positive results (Figure 3). Of note, although LAG3 showed an FDR > 10% when evaluated alone (as shown in Figure 2), when included in the signature the whole signature showed an FDR = 1%, demonstrating the consistency of the results.

**Table 2:** ROC analysis results of all the potential combinations for those genes that predicted individually a statistical significance response to chemotherapy in terms of recurrence at 5 years.

	**Relapse free survival (RFS)**	
	**TNBC (*****n*****:164)**	
**Gene symbol**	**AUC**	***p*-value**
IDO1 + CXCL9	0.678	8.10E-05
IDO1 + CXCL10	0.647	1.16E-03
IDO1 + HLA-DRA	0.634	3.10E-03
IDO1 + IRF9	0.631	3.89E-03
IDO1 + CXCL13	0.665	2.69E-04
IDO1 + HLA-E	0.647	1.13E-03
IDO1 + LAG3	0.628	4.60E-03
IDO1 + STAT1	0.696	1.50E-05
IDO1 + GZMB	0.634	2.99E-03
CXCL9 + CXCL10	0.663	3.21E-04
CXCL9 + HLA-DRA	0.660	4.00E-04
CXCL9 + IRF9	0.682	5.50E-05
CXCL9 + CXCL13	0.672	1.42E-04
CXCL9 + HLA-E	0.671	1.56E-04
CXCL9 + LAG3	0.675	1.09E-04
CXCL9 + STAT1	0.697	1.40E-05
CXCL9 + GZMB	0.672	1.45E-04
CXCL10 + HLA-DRA	0.649	9.51E-04
CXCL10 + IRF9	0.655	5.94E-04
CXCL10 + CXCL13	0.656	5.59E-04
CXCL10 + HLA-E	0.650	9.46E-04
CXCL10 + LAG3	0.646	1.28E-03
CXCL10 + STAT1	0.677	9.00E-05
CXCL10 + GZMB	0.644	1.46E-03
HLA-DRA + IRF9	0.624	6.32E-03
HLA-DRA + CXCL13	0.635	2.85E-03
HLA-DRA + HLA-E	0.621	7.34E-03
HLA-DRA + LAG3	0.620	7.74E-03
HLA-DRA + STAT1	0.666	2.35E-04
HLA-DRA + GZMB	0.627	4.94E-03
IRF9 + CXCL13	0.662	3.53E-04
IRF9 + HLA-E	0.632	3.56E-03
IRF9 + LAG3	0.615	1.12E-02
IRF9 + STAT1	0.694	1.90E-05
IRF9 + GZMB	0.644	1.46E-03
CXCL13 + HLA-E	0.649	9.51E-04
CXCL13 + LAG3	0.666	2.34E-04
CXCL13 + STAT1	0.687	3.50E-05
CXCL13 + GZMB	0.656	5.79E-04
HLA-E + LAG3	0.622	7.09E-03
HLA-E + STAT1	0.688	3.30E-05
HLA-E + GZMB	0.631	3.85E-03
LAG3 + STAT1	0.694	1.70E-05
LAG3 + GZMB	0.619	8.25E-03
STAT1 + GZMB	0.694	1.70E-05
IDO1 + STAT1 + GZMB	0.696	1.40E-05
**CXCL9** **+** **STAT1** **+** **GZMB**	0.697	1.30E-05
CXCL10 + STAT1 + GZMB	0.675	1.11E-04
HLA-DRA + STAT1 + GZMB	0.670	1.74E-04
IRF9 + STAT1 + GZMB	0.693	2.00E-05
CXCL13 + STAT1 + GZMB	0.689	3.00E-05
HLA-E + STAT1 + GZMB	0.689	2.90E-05
LAG3 + STAT1 + GZMB	0.695	1.60E-05
**IDO1** **+** **STAT1** **+** **GZMB** **+** **CXCL9**	0.699	1.10E-05
IDO1 + STAT1 + GZMB + CXCL10	0.678	8.70E-05
IDO1 + STAT1 + GZMB + HLA-DRA	0.676	9.80E-05
IDO1 + STAT1 + GZMB + IRF9	0.692	2.10E-05
IDO1 + STAT1 + GZMB + CXCL13	0.694	1.90E-05
IDO1 + STAT1 + GZMB + HLA-E	0.690	2.50E-05
**IDO1** **+** **STAT1** **+** **GZMB** **+** **LAG3**	0.697	1.40E-05
**IDO1** **+** **STAT1** **+** **GZMB** **+** **LAG3** **+** **CXCL9**	0.699	1.10E-05
IDO1 + STAT1 + GZMB + LAG3 + CXCL10	0.678	8.70E-05
IDO1 + STAT1 + GZMB + LAG3 + HLA-DRA	0.677	9.30E-05
IDO1 + STAT1 + GZMB + LAG3 + IRF9	0.692	2.10E-05
IDO1 + STAT1 + GZMB + LAG3 + CXCL13	0.694	1.80E-05
IDO1 + STAT1 + GZMB + LAG3 + HLA-E	0.690	2.60E-05
IDO1 + STAT1 + GZMB + LAG3 + HLA-DRA + CXCL9	0.685	4.30E-05
IDO1 + STAT1 + GZMB + LAG3 + HLA-DRA + CXCL10	0.678	8.70E-05
IDO1 + STAT1 + GZMB + LAG3 + HLA-DRA + IRF9	0.680	7.20E-05
IDO1 + STAT1 + GZMB + LAG3 + HLA-DRA + HLA-E	0.674	1.20E-04
IDO1 + STAT1 + GZMB + LAG3 + HLA-DRA + CXCL13	0.681	6.20E-05
IDO1 + STAT1 + GZMB + LAG3 + HLA-DRA + CXCL9 + CXCL10	0.681	6.20E-05
IDO1 + STAT1 + GZMB + LAG3 + HLA-DRA + CXCL9 + IRF9	0.687	3.50E-05
IDO1 + STAT1 + GZMB + LAG3 + HLA-DRA + CXCL9 + HLA-E	0.683	5.30E-05
IDO1 + STAT1 + GZMB + LAG3 + HLA-DRA + CXCL9 + CXCL13	0.690	2.50E-05
IDO1 + STAT1 + GZMB + LAG3 + HLA-DRA + CXCL9 + CXCL13 + CXCL10	0.686	4.00E-05
IDO1 + STAT1 + GZMB + LAG3 + HLA-DRA + CXCL9 + CXCL13 + IRF9	0.689	2.90E-05
IDO1 + STAT1 + GZMB + LAG3 + HLA-DRA + CXCL9 + CXCL13 + HLA-E	0.687	3.60E-05
IDO1 + STAT1 + GZMB + LAG3 + HLA-DRA + CXCL9 + CXCL13 + IRF9 + CXCL10	0.687	3.50E-05
IDO1 + STAT1 + GZMB + LAG3 + HLA-DRA + CXCL9 + CXCL13 + IRF9 + HLA-E	0.688	3.30E-05
IDO1 + STAT1 + GZMB + LAG3 + HLA-DRA + CXCL9 + CXCL13 + IRF9 + HLA-E + CXCL10	0.685	4.10E-05

*The combinations more closely related to better chemotherapy response are displayed in orange-red colors; the combinations related to a better chemotherapy response to a lesser extent are displayed in yellow-green colors*.

### The Immunologic Transcriptomic Signature Is Predictive Only in Triple Negative Breast Cancer

**Figure 4:**
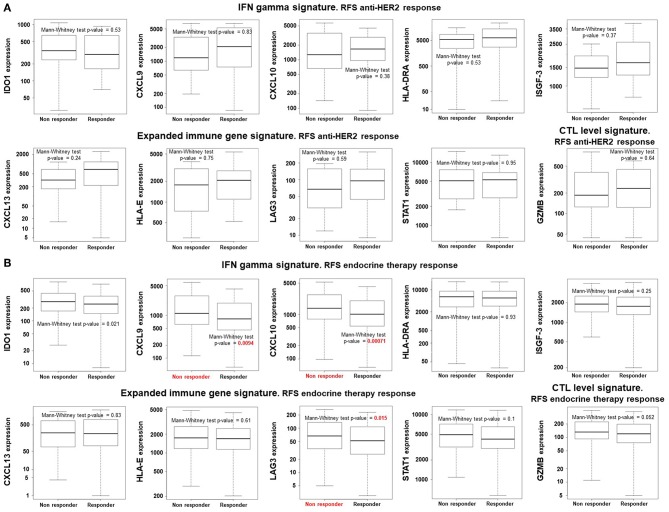
Expression of immune related gene does not predict better outcome (RFS) in patients treated with anti-HER2 or endocrine therapies. Box-plots comparing responders (relapse free survival) vs. non-responders using a Mann-Whitney test in anti-HER2 **(A)** and endocrine therapy **(B)** treated breast cancer patients. All genes have an FDR > 10%, except for CXCL9 and CXCL10.

Finally, we aimed to explore if the identified genes were able to predict RFS to other treatment modalities, including anti-HER2 therapies in combination with chemotherapies or to endocrine treatment alone. Figure 4A shows no association of any of the genes with response for anti-HER2 containing treatments. In case of endocrine therapies, for these genes no association with better response was found. In contrast, expression of CXCL9, CXCL10, and LAG3 predicted detrimental RFS (Figure 4B). These data demonstrate that the predictive capability is observed in TNBC tumors receiving only chemotherapy.

## Discussion

In the present article, based on established immunologic signatures, we identified genes that are able to predict RFS to chemotherapy in basal-like breast cancer patients. Globally those tumors with an immune-surveillance state have a more favorable prognosis and are considered to respond better to check point inhibitors (12, 13). In addition, some tumor subtypes have an immune activated state compared to others, and this is the special case of basal-like breast tumors that do respond to immunotherapy (4).

Here, we identify immunologic genes that do predict RFS following chemotherapy in the neoadjuvant setting and favorable outcome in early stage TNBC breast cancers. To do so, we used recognized immunologic signatures that have shown to predict outcome for checkpoint inhibitors (10, 11). Among them we included the IFN gamma signature, the expanded immune signature, the CTL signature, and the expression of HLA A and HLA B (10, 11). Some of them include CXC chemokines that are mainly secreted in response to INF-γ like CXCL9 and CXCL10 with the main objective of attracting effector T cells, and CXCL13 is a B cell chemoattractant (15). The expression of antigen recognition molecules, like HLA-DRA or HLA-E, was also found to be associated with response. This finding suggests the importance of CD4+T cells in immune response (16), and the recognition of natural killer cells, as is the case for HLA-E (17). STAT1 is an intracellular signaling mediator that can translocate to the nucleus and act as a transcription factor (18). It is an important transcription factor for the Th1 functional orientation of helper T cells. It is activated by multiple ligands including type I interferons (19), and forms a dimer binding with the stimulated gene factor 3 complex (ISGF-3), and enters the nucleus (18). In our analysis we have included additional genes and transcription factors beyond the genes already included in the selected signatures, but only known targets like ICOS, TIGIT, CTLA-4, and CD274 showed association with relapse. LAG3 is mainly expressed on activated T cells and negatively regulate their activation; being a marker of exhaustion (20). GMZB is a serine protease released by cytotoxic T cells that induces apoptosis (21) and Indoleamine 2,3-dioxygenase has been implicated in the modulation of the T cell response (22). Of note, the marker in our analysis, with the uppermost capability to predict outcome in TNBC patients treated with chemotherapy, was STAT1; indicative of the importance of this route in the activation of the immune response. For the prediction of outcome in patients with early stage disease, the combination of IDO1, LAG3, STAT1, and GZMB predicted the best prognosis compared to other signatures or individual genes. Of note this combination showed an FDR = 1%, demonstrating the consistency of the results.

We recognized that several gene signatures have been developed in relation with response to immunotherapies, as the ones used in this article; and others have been described to be only associated with prognosis (10, 11, 23). However, in this work we demonstrate that tumors with a pre-existing stage of immunologic activation are those that do respond better to chemotherapy and have a favorable prognosis in TNBC only. We also acknowledge that other gene signatures could be used or selected for this study. These results are relevant and can help to select those patients that would respond more efficiently to chemotherapy. Furthermore, one could suggest that patients with these markers might benefit from treatment with immunotherapy in addition to chemotherapy. To address this last point, we have initiated an ongoing study to explore the role of these genes in predicting response to chemotherapy in combination with checkpoint inhibitors.

Finally, we observed that expression of these genes did not predict pCR, suggesting that this non-time to event endpoint is not a good surrogate marker to detect long term outcome for immune activated tumors.

A relevant finding of our study is the association identified between the described genes and the high expression of CD8 cells using the EPIC tool (14), in tumors that did not relapse within the first 5 years, confirming the importance of the T cell response in the long term outcome, and particularly in patients treated with chemotherapy. In line with our study, a recent article has evaluated gene expression signatures with immune infiltrates using laser microdissection and describing several gene signatures in TNBC (24).

In brief, we identified a focused immunologic gene signature that predicts the outcome to chemotherapy in neoadjuvant TNBC patients. Studies aiming to confirm their value are ongoing.

## Data Availability Statement

The datasets generated for this study are available on request to the corresponding author.

## Author Contributions

AO conceived the study and created the original design of the experiments. JP-P, JT, RP, MB-P, JG-S, VG-B, AM, and PP-S searched for the data used and performed the analysis. AO, JP-P, BG, AE-O, AP, and MB-P wrote the manuscript. All authors reviewed, modified, and approved the final version of the manuscript.

### Conflict of Interest

AE-O receives research funding from Entrechem, travel expenses from Merck, and advisory board fees from Daiichi Sankyo. JG-S receives consultancy/speaker fees from Novartis, Celgene, Eli Lilly, EISAI, Roche; institution research funding from Astrazeneca; and travel support from Novartis, Roche, and Pfizer. AP receives consultancy fees from Daiichi Sankyo. The remaining authors declare that the research was conducted in the absence of any commercial or financial relationships that could be construed as a potential conflict of interest. The reviewer FP and handling Editor declared their shared affiliation at the time of review.
