# Supplementary material for: A Transcriptomic Immunologic Signature Predicts Favorable Outcome in Neoadjuvant Chemotherapy Treated Triple Negative Breast Tumors
Source: Front Immunol. 2019 Dec 18;10:2802. doi: 10.3389/fimmu.2019.02802 (PMC6930158; doi:10.3389/fimmu.2019.02802)
Supplement: Supplementary file 1 [file Data_Sheet_1.pdf]

Supplementary Material

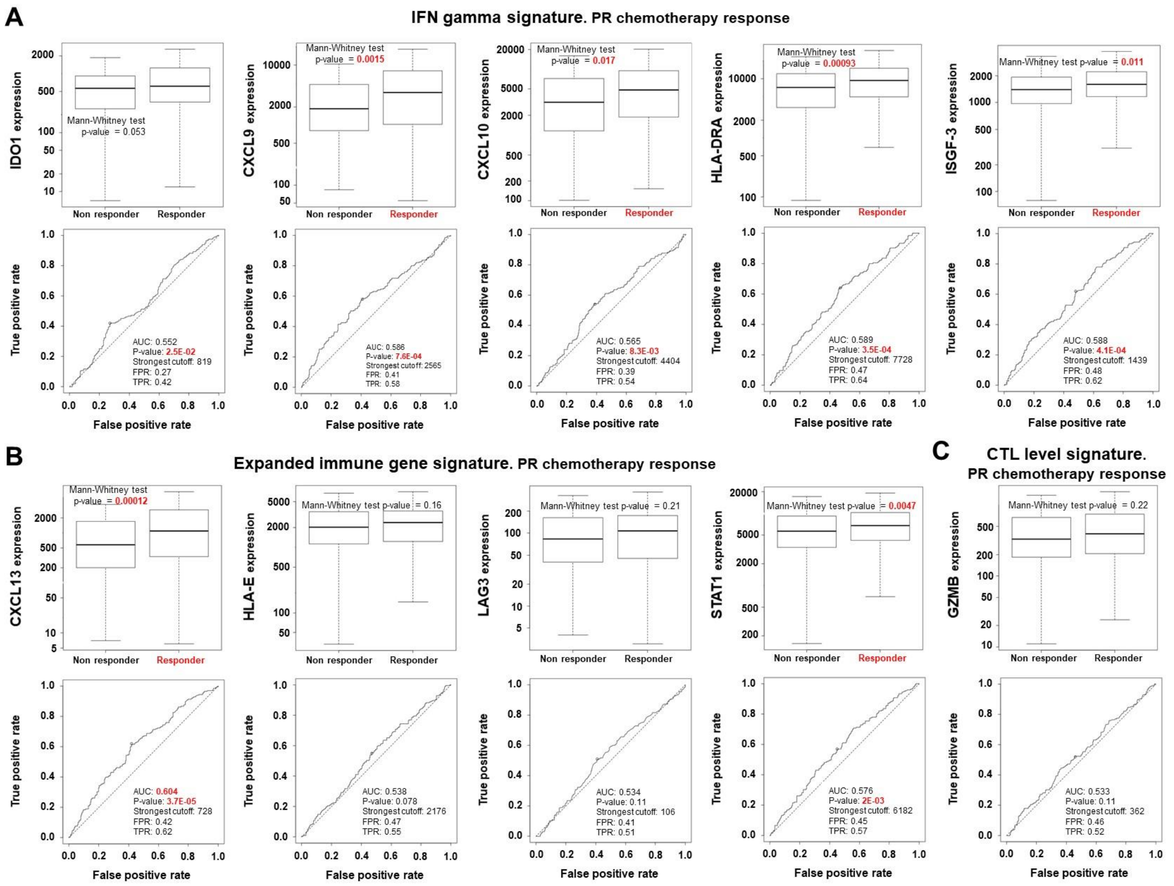

Supplementary Figure 1. Expression of immune related response genes does not predict pathological complete response (pCR) in chemotherapy treated TNBC patients. Box-plots comparing responders (pCR) versus non-responders using a Mann-Whitney test and Area Under the Curve (AUC) (p values, the strongest cutoff, the False Positive Rate (FPR), and the True Positive Rate (TPR)) were calculated for each gene for A. INF gamma signature B. Expanded immune gene signature C. CTL level signature.

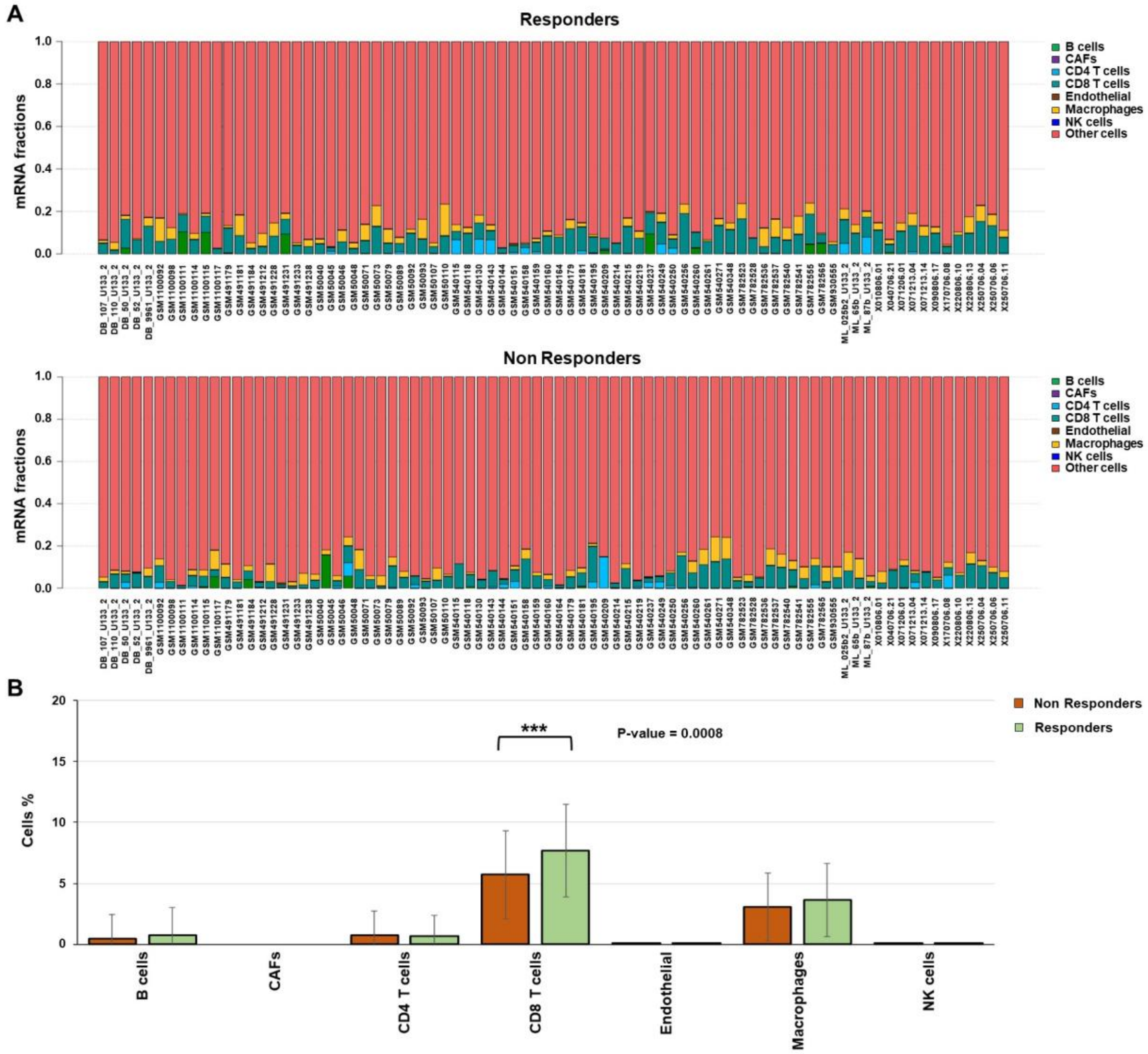

Supplementary Figure 2. Tumor microenvironment composition analyzed by EPIC tool. (A) Tumor microenvironment composition evaluating our own gene signature: IDO1, CXCL9, CXCL10, CXCL13, HLA-DRA, HLA-E, IRF9, LAG3, STAT1 and GZMB, in every TNBC patient sample divided by Responders and Non-Responders to any Chemotherapy treatment. (B) Median percentage values of every immunological population in both Responders and Non-Responders to any Chemotherapy treatment TNBC patients evaluating our own gene signature.

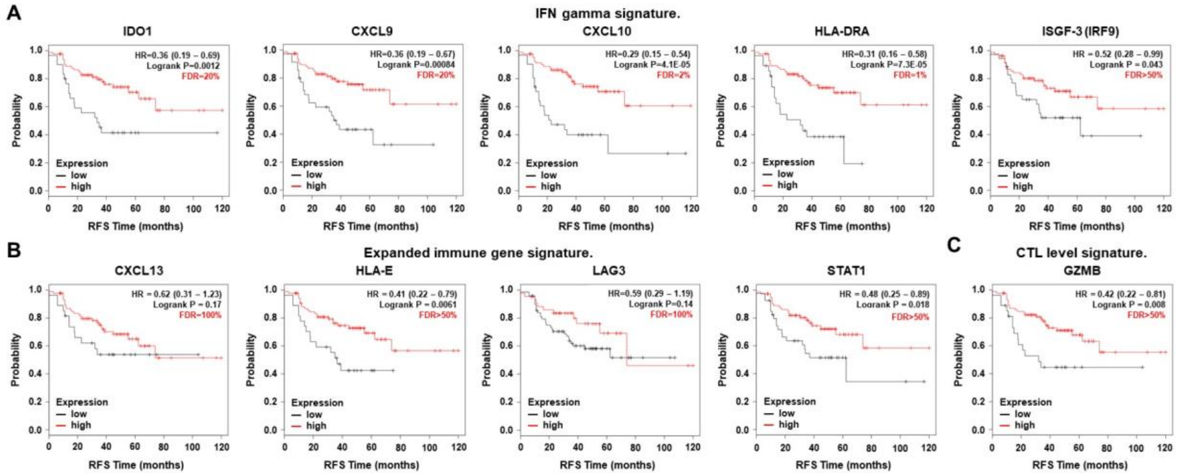

Supplementary Figure 3. The outcome (RFS) prediction for early stage chemotherapy treated TNBC patients with overexpression of our immune related signature genes. Kaplan-Meier plots show survival differences. **(A)**, INF gamma signature **(B)**, Expanded immune gene signature **(C)**, CTL level signature.

Supplementary Table 1. Patients' clinical data.

GEO sample ID	Neoadjuvant_chemotherapy	ER_status	HER2_status	Lymph node status	Grade	Molecular subtype - StGallen (1=TNBC, 2=luminal A(ER+HER2-KI67-), 3=luminal B, 4=HER2+ER-)	Age
GSM411292	1	0	1	1	3	4	
GSM411293	1	0	1	1	3	4	
GSM411294	1	0	1	0	3	4	
GSM411295	1	0	1	1	3	4	
GSM411297	1	0	0	0	3	1	
GSM411298	1	0	1	1	3	4	
GSM411299	1	0	1	1	3	4	
GSM411300	1	0	1	0	3	4	
GSM411301	1	0	1	0	3	4	
GSM411302	1	0	1	0	2	4	
GSM411303	1	0	0	1	3	1	
GSM411304	1	1	1	1	2	3	
GSM411305	1	0	1	1	3	4	
GSM411306	1	0	0	1	3	1	
GSM411307	1	0	1	1	3	4	
GSM411308	1	0	0	0	3	1	
GSM411309	1	0	0	1	3	1	
GSM411310	1	0	0	1	3	1	
GSM411311	1	0	0	1	3	1	
GSM411312	1	0	0	1	3	1	
GSM411313	1	0	0	0	3	1	
GSM411314	1	0	0	1	2	1	
GSM411315	1	0	0	0	2	1	
GSM411316	1	0	1	1		4	
GSM411317	1	0	0	1	3	1	
GSM411318	1	0	0	0	3	1	
GSM411319	1	1	0	1	3	3	
GSM411320	1	0	0	0	2	1	

GSM411321	1	0	1	1	3	4	
GSM411322	1	0	0	1	3	1	
GSM411323	1	1	0	1	2	2	
GSM411324	1	0	0	1	3	1	
GSM411325	1	0	0	1	2	1	
GSM411326	1	0	0	1	3	1	
GSM411327	1	0	0	0	3	1	
GSM411328	1	0	0	0	3	1	
GSM411329	1	0	0	1	3	1	
GSM411330	1	0	1	0	2	4	
GSM411331	1	0	0	0	3	1	
GSM411332	1	0	0	1	3	1	
GSM411333	1	0	0	0	3	1	
GSM411334	1	0	0	0	3	1	
GSM411335	1	0	0	0	3	1	
GSM411336	1	0	0	0		1	
GSM411337	1	0	0	0	3	1	
GSM411338	1	0	1	0	3	4	
GSM411339	1	0	0	1	2	1	
GSM411340	1	0	0	1	3	1	
GSM411341	1	0	0	0	2	1	
GSM411342	1	0	0	0	3	1	
GSM411343	1	0	0	0	2	1	
GSM411344	1	0	0	1	3	1	
GSM411345	1	0	0	1	1	1	
GSM411346	1	0	0	0	3	1	
GSM411347	1	0	0	0	3	1	
GSM411348	1	0	0	1	3	1	
GSM411349	1	0	0	0	2	1	
GSM411350	1	0	0	0	3	1	
GSM411351	1	0	1	1	3	4	
GSM411352	1	0	0	0	3	1	
GSM411353	1	0	0	0	3	1	
GSM411354	1	0	0	1	3	1	
GSM411355	1	0	0	0	3	1	
GSM411356	1	0	0	0	2	1	
GSM411357	1	0	0	1	3	1	
GSM411358	1	0	0	0	2	1	
GSM411359	1	0	0	1	3	1	
GSM411360	1	0	0	1	3	1	
GSM411361	1	0	0	0	3	1	
GSM411362	1	0	0	0	2	1	
GSM411363	1	0	1	0	3	4	
GSM411365	1	0	0	0	3	1	
GSM411366	1	0	1	1	3	4	

GSM411368	1	0	0	1	3	1	
GSM411369	1	0	1	1	3	4	
GSM411370	1	0	0	0	3	1	
GSM411371	1	0	0	1	3	1	
GSM411372	1	0	0	1	3	1	
GSM411373	1	0	0	0	3	1	
GSM411374	1	0	0	1	3	1	
GSM411375	1	0	0	0	3	1	
GSM411376	1	0	0	1	3	1	
GSM411377	1	0	0	1	3	1	
GSM411378	1	0	0	0	3	1	
GSM411379	1	0	0	0	3	1	
GSM411380	1	0	0	0	3	1	
GSM411381	1	0	0	1	3	1	
GSM411382	1	1	0	0	3	2	
GSM411383	1	0	0	1	2	1	
GSM411384	1	0	0	1	3	1	
GSM411385	1	0	0	0	1	1	
GSM411386	1	0	0	1	3	1	
GSM411387	1	0	0	1	3	1	
GSM411388	1	0	0	0	2	1	
GSM411389	1	0	0	1	3	1	
GSM411390	1	0	1	1	3	4	
GSM411391	1	0	0	0		1	
GSM411392	1	0	0	1		1	
GSM505377	1	0	1	1	3	4	58
GSM505379	1	1	0	1	2	2	53
GSM505381	1	0	1	1	3	4	39
GSM505383	1	1	0	1	3	2	42
GSM505385	1	1	1	1	3	3	51
GSM505389	1	1	1	1	3	3	62
GSM505393	1	1	1	1	3	3	71
GSM505395	1	1	1	1	3	3	52
GSM505397	1	1	0	0	2	3	54
GSM505399	1	1	0	1	3	2	45
GSM505401	1	0	1	1	3	4	53
GSM505403	1	1	0	0	2	2	52
GSM505405	1	1	1	0	2	3	73
GSM505407	1	1	0	1	3	2	79
GSM505409	1	1	0	0	3	3	67
GSM505440	1	1	0	1	3	2	64
GSM505441	1	1	1	0	3	3	65
GSM505442	1	1	0	1	3	2	67
GSM505443	1	1	1	0	3	3	28
GSM505444	1	1	0	0	2	2	42

GSM505446	1	1	0	1	2	2	35
GSM505470	1	0	0	1		1	40
GSM505489	1	0	0	1		1	35
GSM505490	1	0	0	1		1	42
GSM505491	1	1	0	1	3	3	50
GSM505492	1	1	0	1		3	52
GSM505493	1	1	0			2	
GSM505494	1	1	1			3	46
GSM505495	1	1	0		3	3	50
GSM505497	1	1	0			3	41
GSM505498	1	1	0			3	46
GSM505499	1	0	0		3	1	36
GSM505500	1	0	0	1	3	1	46
GSM505501	1	1	0			3	61
GSM505502	1	1	0			3	46
GSM505503	1	0	0	1	3	1	60
GSM505504	1	0	0	1	3	1	51
GSM505505	1	0	1	1	3	4	50
GSM508013	1	1	0	0	2	2	55
GSM508014	1	1	0	0	3	3	45
GSM508016	1	1	0	0	1	3	49
GSM508017	1	0	1	1	2	4	39
GSM508018	1	1	0	1	3	3	48
GSM508019	1	0	0	0	3	1	73
GSM508022	1	1	0	1	3	3	45
GSM508023	1	1	0	0	2	3	62
GSM508026	1	1	0	1	2	3	55
GSM508028	1	1	0	1	1	3	45
GSM508029	1	0	1	0	3	4	73
GSM508030	1	1	0	0	2	2	50
GSM508031	1	1	0	1	2	3	50
GSM508032	1	1	0	0	2	2	64
GSM508033	1	1	0	1		2	43
GSM508034	1	1	0	1	3	3	43
GSM508039	1	1	0	1	3	3	32
GSM508040	1	1	1	1	2	3	54
GSM508041	1	1	0	1	2	3	66
GSM508043	1	0	0	1	3	1	58
GSM508049	1	0	0	1	3	1	42
GSM508051	1	1	0	1	3	2	62
GSM508052	1	0	0	0	3	1	64
GSM508056	1	1	0	1	3	3	40
GSM508058	1	0	0	1		1	74
GSM508060	1	1	1	1	2	3	52
GSM508061	1	1	0	0		3	42

GSM508062	1	1	1	1	2	3	45
GSM508063	1	1	0	1		3	57
GSM508064	1	1	0	0		3	61
GSM508065	1	1	0	1		3	56
GSM508066	1	0	0	1		1	34
GSM508067	1	1	0	0	3	3	49
GSM508068	1	0	0	1		1	68
GSM508069	1	0	0	1		1	36
GSM508070	1	0	1	1		4	49
GSM508072	1	0	1	0	3	4	49
GSM508073	1	1	0	0	3	2	60
GSM508074	1	1	0	0	3	3	56
GSM508075	1	1	0	1	2	2	44
GSM508076	1	1	0	0	2	2	47
GSM508077	1	1	0		2	2	45
GSM508078	1	1	1	0	3	3	59
GSM508079	1	0	0	1	3	1	53
GSM508080	1	0	0	1	3	1	46
GSM508081	1	1	0	0	2	3	40
GSM508082	1	1	0	1	2	3	48
GSM508083	1	1	0	0	1	3	61
GSM508084	1	1	0	0	2	2	62
GSM508085	1	0	0	1	3	1	46
GSM508086	1	0	1	1	2	4	47
GSM508088	1	1	0	0	2	3	49
GSM508089	1	0	0	1	3	1	38
GSM508090	1	1	0	0	3	3	66
GSM508091	1	0	0	1	3	1	31
GSM508092	1	1	0	1	3	3	44
GSM508093	1	0	0	0	2	1	52
GSM508094	1	1	0	1	2	3	43
GSM508095	1	1	0	1	2	3	34
GSM508096	1	1	0	1	2	2	48
GSM508097	1	1	0	0	2	3	57
GSM508098	1	1	0	0	2	3	73
GSM508101	1	1	0	0	2	2	66
GSM508102	1	1	0	1	2	2	47
GSM508108	1	1	0	1	1	2	66
GSM508109	1	1	0	0	1	2	45
GSM508113	1	1	0	0	3	3	31
GSM508118	1	1	0	1	3	3	42
GSM508123	1	1	0	1	3	2	40
GSM508131	1	1	0	0	3	3	53
GSM508134	1	1	0	1	3	3	43
GSM508135	1	1	1	1	3	3	37

GSM508141	1	1	0	1		3	53
GSM508143	1	1	0	1	2	2	48
GSM508148	1	0	1	1	3	4	50
GSM508153	1	1	0	1		3	37
GSM508154	1	1	0	0	3	3	61
GSM508155	1	1	0	1	3	3	61
GSM508156	1	0	1	1	3	4	46
GSM508157	1	0	0	0		1	65
GSM508160	1	0	0	1		1	44
GSM508161	1	1	0	1		3	29
GSM508162	1	0	0	1		1	55
GSM508168	1	1	0	0	2	2	67
GSM809184	1	1	0	1	3	3	46
GSM809186	1	0	0	1	2	1	67
GSM809187	1	1	1	1	2	3	56
GSM809188	1	1	0	1	2	2	40
GSM809189	1	1	0	1	2	2	57
GSM809190	1	1	1	1	2	3	48
GSM809191	1	1	0	0	1	2	49
GSM809193	1	1	1	0	2	3	45
GSM809195	1	1	0	0	2	2	58
GSM809196	1	1	0	1	2	2	33
GSM809197	1	1	0	1	1	2	44
GSM809198	1	1	0	1	2	3	51
GSM809199	1	1	1	1	2	3	46
GSM809200	1	1	0	1	2	3	48
GSM809201	1	1	0	1	2	3	46
GSM809204	1	0	0	0	2	1	30
GSM809205	1	1	0	0	2	2	40
GSM809206	1	1	0	1	2	2	35
GSM809207	1	1	0	1	1	2	59
GSM809208	1	1	0	1	2	2	50
GSM809209	1	0	0	1	3	1	43
GSM809210	1	1	0	1	2	2	49
GSM809211	1	0	1	1	3	4	65
GSM809212	1	1	1	1	2	3	31
GSM809214	1	1	0	1	2	2	42
GSM809215	1	1	0	0	2	3	33
GSM809216	1	1	0	1	2	2	56
GSM809217	1	1	0	1	2	2	56
GSM809218	1	1	0	0	2	2	43
GSM809219	1	1	0	1	2	3	60
GSM809220	1	0	0	1	1	1	58
GSM809221	1	0	1	1	3	4	48
GSM809222	1	1	0	0	2	3	72

GSM809223	1	1	0	1	3	3	52
GSM809224	1	0	0	0	2	1	63
GSM809227	1	0	0	1	2	1	49
GSM809228	1	1	0	0	2	2	59
GSM809230	1	1	0	0	2	2	42
GSM809233	1	1	0	1	2	3	47
GSM809234	1	1	0	1	2	2	57
GSM809235	1	1	0	1	3	2	57
GSM809239	1	0	0	1	2	1	68
GSM809240	1	1	0	0	2	2	45
GSM809241	1	1	0	0	2	3	27
GSM809242	1	1	0	0	2	2	58
GSM809243	1	1	1	0	2	3	37
GSM809244	1	1	0	1	1	2	39
GSM809245	1	0	1	1	2	4	44
GSM809247	1	0	0	1	3	1	54
GSM809249	1	1	0	1	2	3	54
GSM809250	1	1	0	0	2	3	36
GSM809251	1	1	0	1	2	2	61
GSM809252	1	1	1	0	3	3	43
GSM809254	1	1	0	1	2	2	47
GSM809255	1	1	0	1	3	2	62
GSM809257	1	1	0	1	1	2	48
GSM809258	1	1	0	0	2	2	59
GSM809259	1	1	0	0	1	2	48
GSM809260	1	1	0	0	1	2	48
GSM809261	1	1	0	1	2	3	70
GSM809262	1	1	0	1	2	2	48
GSM809263	1	1	0	1	2	2	49
GSM809264	1	0	1	1	2	4	57
GSM809265	1	1	0	1	2	2	54
GSM809267	1	0	1	1	3	4	51
GSM809268	1	1	0	1	1	2	63
GSM809269	1	1	0	1	2	3	42
GSM809270	1	1	1	1	1	3	66
GSM809273	1	0	0	1	3	1	63
GSM809275	1	1	0	1	2	2	73
GSM809276	1	0	0	1	2	1	55
GSM809277	1	0	0	0	3	1	36
GSM809278	1	0	0	1	2	1	63
GSM809279	1	1	0	0	2	3	48
GSM809281	1	1	0	0	3	2	60
GSM809282	1	0	0	1	2	1	39
GSM809283	1	1	0	1	2	2	56
GSM809284	1	1	0	1	2	2	40

GSM809285	1	1	0	1	2	2	68
GSM809286	1	1	0	0	1	2	49
GSM809287	1	1	0	0	2	3	55
GSM809288	1	1	0	1	2	2	45
GSM809290	1	1	0	1	1	2	44
GSM809291	1	1	0	0	2	2	37
GSM809292	1	1	0	0	2	2	47
GSM809293	1	1	0	1	1	2	56
GSM809296	1	1	0	1	1	2	42
GSM809297	1	1	0	0	2	2	68
GSM465195	1	0	0			1	
GSM465200	1	1	0			2	
GSM465205	1	1	0			2	
GSM465211	1	0	0			1	
GSM465217	1	1	0			2	
GSM465220	1	1	0			3	
GSM465226	1	1	0			2	
GSM465234	1	1	0			2	
GSM465239	1	0	0			1	
GSM465187	1	1	0			2	
GSM465192	1	1	0			2	
GSM465193	1	1	0			3	
GSM465196	1	1	0			2	
GSM465197	1	1	0			2	
GSM465202	1	0	0			1	
GSM465207	1	0	0			1	
GSM465208	1	0	0			1	
GSM465212	1	1	0			2	
GSM465218	1	1	0			2	
GSM465219	1	1	0			2	
GSM465227	1	1	1			3	
GSM465229	1	1	0			3	
GSM465232	1	1	0			2	
GSM465238	1	1	0			2	
GSM465241	1	0	1			4	
GSM590840	1	1	0	0		3	46.209
GSM590847	1	1	0	1	2	3	47.118
GSM590867	1	0	0	1	3	1	66.82
GSM590877	1	1	0	2	3	3	33.539
GSM590887	1	1	0	1	2	3	47.899
GSM590899	1	1	0	2	2	2	58.13
GSM590900	1	1	0	1	3	2	44.225
GSM1030093	1	0	1			4	40
GSM1030098	1	1	0			2	40
GSM1030107	1	1	0			2	51

GSM1030108	1	1	0			2	50
GSM1030111	1	1	0			2	47
GSM1030112	1	1	0			2	66
GSM1030117	1	1	0			2	41
GSM1030120	1	1	0			2	37
GSM1030124	1	1	0			2	54
GSM1030125	1	1	0			2	50
GSM1030129	1	1	0			2	42
GSM1030130	1	1	0			2	40
GSM1030133	1	1	0			2	29
GSM1030134	1	1	0			2	60
GSM1030135	1	1	0			2	56
GSM1030137	1	1	0			2	46
GSM1030143	1	1	0			2	43
GSM1030148	1	1	0			2	41
GSM1030154	1	1	0			2	70
GSM1030161	1	0	0			1	57
GSM1030165	1	0	0			1	52
GSM1030178	1	1	0			3	30
GSM1030181	1	0	0			1	61
GSM1030184	1	0	0			1	62
GSM1030197	1	0	0			1	58
GSM1030208	1	0	0			1	51
GSM1030211	1	0	0			1	55
GSM1030214	1	0	0			1	67
GSM1030216	1	0	0			1	63
GSM1030220	1	1	0			2	42
GSM1030227	1	1	0			2	58
GSM1030231	1	0	1			4	45
GSM1030232	1	0	1			4	39
GSM1030237	1	0	1			4	53
GSM1030239	1	0	1			4	56
GSM1030244	1	1	0			2	60
GSM1030245	1	1	0			2	55
GSM1030246	1	1	0			2	42
GSM1030252	1	1	0			2	43
GSM1030254	1	1	0			2	45
GSM1030255	1	1	0			2	57
GSM1030256	1	1	0			2	55
GSM1030258	1	1	0			2	58
GSM1030259	1	1	0			2	39
GSM1030268	1	1	0			3	36
GSM1030269	1	1	0			2	44
GSM1030283	1	1	0			2	74
GSM1030284	1	1	0			2	39

GSM1030286	1	0	0			1	37
GSM1030295	1	0	1			4	54
GSM1030300	1	0	0			1	36
GSM1030302	1	0	0			1	48
GSM1030307	1	0	0			1	52
GSM1030308	1	0	0			1	52
GSM1030309	1	0	0			1	52
GSM1030313	1	0	0			1	37
GSM1030320	1	0	0			1	57
GSM1030325	1	0	0			1	42
GSM1030326	1	1	0			2	40
GSM1030328	1	0	0			1	52
GSM1030335	1	0	0			1	69
GSM1030336	1	0	0			1	26
GSM1030346	1	0	0			1	34
GSM1030347	1	0	0			1	59
GSM1030354	1	1	0			2	62
GSM505367	1	1	1		3	3	39
GSM505369	1	0	0		3	1	59
GSM505362	1	1	0		3	3	51
GSM505482	1	1	1		2	3	45
GSM505329	1	1	1		2	3	77
GSM505340	1	0	1		3	4	54
GSM505376	1	0	1		3	4	58
GSM505400	1	0	1	0	3	4	53
GSM505384	1	1	1		3	3	51
GSM505394	1	1	1		3	3	52
GSM505396	1	1	0		2	2	54
GSM505414	1	1	1		3	3	79
GSM505406	1	1	0		3	2	79
GSM505404	1	1	1		2	3	73
GSM505380	1	0	1	1	3	4	39
GSM505388	1	1	1	1	3	3	62
GSM505417	1	0	0	1	3	1	51
GSM505418	1	0	1	1	3	4	55
GSM505425	1	0	1		3	4	50
GSM505431	1	1	1		3	3	65
GSM505433	1	1	0		2	2	35
GSM505522	1	1	0		2	3	65
GSM505429	1	1	1		3	3	28
GSM505461	1	1	1		3	3	47
GSM505484	1	1	0		2	2	55
GSM505546	1	1	1	1	3	3	70
GSM505555	1	0	0		3	1	45
GSM505599	1	1	0	1	2	3	49

GSM505604	1	1	0		2	3	45
R5723_U133A	1	1	1		3	3	57
X3276_U133A	1	1	0		3	3	36
X6869_U133A	1	1	0		3	2	48
R5731_U133A	1	1	0		2	2	38
R5664_U133A	1	1	0		2	3	65
X5592_U133A	1	0	1		3	4	65
R5665_U133A	1	1	0		2	2	59
X4690_U133A_2	1	1	0		3	2	52
R5668_U133A	1	1	0		2	2	32
R4325_U133A	1	1	0		2	3	48
R5136C_U133A_3	1	1	0		2	2	59
X7117_U133A	1	1	0		2	2	52
X7115_U133A	1	1	0		3	3	32
R4954_U133A	1	1	1		3	3	43
R5059_U133A	1	1	0		2	2	24
X5690_U133A	1	0	1		3	4	34
R2975_U133A	1	1	0		2	2	76
R5693_U133A	1	0	0		3	1	69
X5666_U133A	1	1	0		2	2	49
R4713_U133A	1	1	0		2	2	50
X3866_U133A	1	1	0		2	3	37
X4503_U133A	1	1	0		3	3	41
X6867d_U133A	1	1	0		3	2	44
R5737_U133A	1	1	0		2	2	30
R5694C_U133A	1	1	0		2	2	62
X6866_U133A	1	0	0		3	1	35
GSM930552	1	0	0	0	3	1	57
GSM930553	1	1	1	0	3	3	28
GSM930554	1	1	1	0	3	3	49
GSM930555	1	0	0	0	3	1	41
GSM930556	1	1	1	0	2	3	47
GSM930558	1	1	0	1	3	2	56
GSM930564	1	1	1	0	3	3	44
GSM930567	1	1	1	1	3	3	48
GSM930549	1	1	1	0	3	3	65
GSM930570	1	1	1	1	2	3	72
GSM930550	1	1	1	0	3	3	30
GSM930551	1	0	1	0	3	4	46
GSM930561	1	0	1	1	3	4	30
GSM930562	1	1	0	0	3	3	61
GSM930565	1	1	0	1	3	3	50
GSM930568	1	1	1	1	2	3	61
GSM930572	1	1	1	1	3	3	24
GSM615096	1	1	0	1	2	3	37.8

GSM615097	1	1	0	1	2	3	45.8
GSM615098	1	1	0	0	3	3	40.7
GSM615100	1	1	0	0	2	3	35.5
GSM615101	1	0	0	0	3	1	52.2
GSM615102	1	1	0	0		3	38.2
GSM615103	1	1	0	1		3	54.2
GSM615104	1	1	0	1		3	46.6
GSM615106	1	0	0	0		1	51.1
GSM615107	1	0	0	1		1	37.8
GSM615108	1	1	0	1	3	3	46.6
GSM615109	1	0	0	1		1	58.8
GSM615111	1	1	0	1		3	50.4
GSM615112	1	1	0	0		3	63.2
GSM615113	1	1	0	1		3	42.8
GSM615114	1	1	0	1	2	3	52.8
GSM615115	1	0	0	1	2	1	44.2
GSM615116	1	1	0	0	3	3	54.2
GSM615117	1	1	0	1	3	3	34.6
GSM615120	1	1	0	0	1	3	53.2
GSM615121	1	1	0	0	3	3	36.5
GSM615124	1	1	0	1	2	3	61
GSM615125	1	1	0	0	2	3	44.1
GSM615126	1	1	0	1	3	3	43.6
GSM615127	1	1	0	0	2	3	63.3
GSM615128	1	1	0	0		3	50.4
GSM615129	1	1	0	0		3	53.5
GSM615130	1	1	0	0	2	3	44.8
GSM615131	1	1	0	1		3	61.9
GSM615132	1	1	0	1	2	3	47.6
GSM615133	1	1	0	1	1	3	51.4
GSM615134	1	1	0	1	1	3	43.1
GSM615135	1	1	0	1	3	3	58.9
GSM615136	1	0	0	1	3	1	60
GSM615138	1	1	0	1	2	3	35.1
GSM615141	1	1	0	1		3	59.2
GSM615142	1	1	0	1		3	51.5
GSM615144	1	1	0	1	2	3	61.8
GSM615145	1	1	0	1	3	3	55.7
GSM615147	1	1	0	0		3	60.1
GSM615148	1	1	0	0	3	3	39.7
GSM615149	1	0	0	1	3	1	44.2
GSM615150	1	1	0	1	2	3	44.4
GSM615151	1	0	0		2	1	44.6
GSM615152	1	0	0	1	3	1	44
GSM615153	1	1	0	0	2	2	64.3

GSM615155	1	1	0	1	3	3	31.5
GSM615156	1	0	0	1	3	1	38.3
GSM615157	1	1	0	0	2	3	45.9
GSM615158	1	1	0	1	1	2	59.6
GSM615159	1	0	0	0		1	44.7
GSM615160	1	0	0	1	3	1	37.7
GSM615161	1	1	0	1	2	3	43.8
GSM615162	1	1	0	1	1	3	42.3
GSM615165	1	1	0	0	2	2	43.4
GSM615166	1	1	0	1	2	3	40.6
GSM615168	1	1	0			3	50.1
GSM615169	1	1	0	0	3	3	47.8
GSM615170	1	1	0	0	2	3	46
GSM615172	1	1	0	0	2	3	59.8
GSM615173	1	1	0	1		3	47.8
GSM615174	1	1	0	1	3	3	42.1
GSM615176	1	1	0	1	1	3	45.5
GSM615177	1	1	0	0		3	50
GSM615178	1	0	0	1	3	1	38.6
GSM615179	1	1	0	0	2	2	56
GSM615180	1	1	0	1	2	2	47
GSM615182	1	0	0	0	3	1	75
GSM615183	1	1	0	1	2	3	34
GSM615184	1	0	0	0	3	1	51
GSM615185	1	0	0	0	3	1	65
GSM615187	1	1	0	0	2	2	66
GSM615188	1	1	0	0	2	3	61
GSM615189	1	0	0	1	3	1	65
GSM615190	1	0	0	0	3	1	37
GSM615192	1	1	0	0	2	3	51
GSM615195	1	1	0	1	3	3	29
GSM615196	1	1	0	0	3	3	61
GSM615197	1	0	0	1	3	1	67
GSM615198	1	1	0	0	2	3	57
GSM615199	1	1	0	1	2	2	38
GSM615200	1	1	0	1	3	3	49
GSM615203	1	1	0	1	2	2	73
GSM615204	1	1	0	0	2	2	46
GSM615205	1	1	0	1	2	2	68
GSM615206	1	1	0	0	3	3	47
GSM615207	1	1	0	0	2	3	42
GSM615208	1	1	0	1	2	2	69
GSM615210	1	1	0	1	2	2	75
GSM615214	1	1	0	1	2	2	54
GSM615216	1	1	0	1	2	2	58

GSM615217	1	1	0	0	2	2	52
GSM615218	1	0	0	1	3	1	56
GSM615219	1	1	0	1	3	3	38
GSM615220	1	1	0	1	3	3	31
GSM615221	1	1	0	1	3	2	38
GSM615222	1	1	0	1	2	2	48
GSM615223	1	0	0	1	3	1	57
GSM615224	1	1	0	0	2	2	52
GSM615225	1	0	0	1	3	1	60
GSM615226	1	0	0	0	1	1	58
GSM615227	1	1	0	1	2	2	52
GSM615228	1	1	0	1	3	2	42
GSM615229	1	1	0	1	2	2	48
GSM615230	1	1	1	0	3	3	39
GSM615231	1	1	0	1	2	2	49
GSM615232	1	1	0	0	2	2	56
GSM615233	1	1	0	1	1	3	72
GSM615234	1	1	0	1	3	3	45
GSM615235	1	1	0	0	3	3	67
GSM615236	1	1	1	1	3	3	71
GSM615237	1	1	0	0	2	2	45
GSM615238	1	1	0	1	2	3	44
GSM615239	1	1	0	1	3	2	46
GSM615241	1	1	0	1	2	2	63
GSM615243	1	1	0	0	2	2	46
GSM615244	1	0	0	1	3	1	51
GSM615245	1	1	0	1	2	2	36
GSM615246	1	1	0	0	2	2	26
GSM615247	1	1	0	1	3	2	40
GSM615248	1	0	0	1	3	1	42
GSM615249	1	1	0	1	3	3	67
GSM615250	1	0	0	1	3	1	63
GSM615251	1	0	0	1	3	1	44
GSM615252	1	1	0	1	2	3	54
GSM615253	1	1	0	1	2	2	41
GSM615254	1	1	0	1	2	2	49
GSM615255	1	0	0	1	3	1	39
GSM615257	1	1	0	1	1	3	59
GSM615258	1	1	0	1	2	2	55
GSM615259	1	1	0	0	2	2	62
GSM615260	1	1	0	1	3	3	56
GSM615261	1	1	0	0	3	2	58
GSM615262	1	1	0	1	1	2	42
GSM615265	1	1	0	1	3	2	63
GSM615266	1	1	0	0	2	2	60

GSM615267	1	1	0	0	2	2	42
GSM615268	1	1	0	1	1	3	35
GSM615269	1	1	0	1	2	2	40
GSM615271	1	0	1	1	2	4	44
GSM615272	1	1	0	1	2	2	63
GSM615273	1	1	0	1	2	2	48
GSM615274	1	0	0	1	2	1	68
GSM615275	1	0	0	1	2	1	60
GSM615276	1	0	0	1	3	1	52
GSM615277	1	1	0	1	2	2	50
GSM615278	1	1	0	0	3	2	47
GSM615279	1	1	0	1	2	3	50
GSM615281	1	1	0	0	2	3	44
GSM615282	1	1	0	1	2	3	60
GSM615283	1	1	0	1	2	2	57
GSM615285	1	1	0	0	3	3	44
GSM615286	1	1	0	1	2	2	62
GSM615287	1	1	0	0	2	3	50
GSM615288	1	0	0	0	3	1	38
GSM615289	1	1	0	1	2	3	46
GSM615290	1	0	0	0	2	1	66
GSM615292	1	1	0	1	2	2	47
GSM615294	1	1	0	0	3	2	45
GSM615295	1	1	0	1	3	2	53
GSM615296	1	1	0	1	2	2	46
GSM615297	1	1	0	1	3	2	58
GSM615298	1	1	0	1	2	3	40
GSM615299	1	1	0	1	2	2	47
GSM615301	1	1	0	1	3	3	62
GSM615302	1	1	0	1	2	3	55
GSM615303	1	0	0	1	3	1	68
GSM615304	1	1	0	1	3	3	57
GSM615305	1	1	0	1	2	2	39
GSM615306	1	1	0	1	2	3	58
GSM615308	1	1	0	1	3	3	51
GSM615309	1	1	0	1	3	3	45
GSM615311	1	1	0	1	2	2	34
GSM615312	1	0	0	1	2	1	51
GSM615313	1	1	0	1	1	3	50
GSM615314	1	1	0	1	2	2	71
GSM615315	1	0	0	1	3	1	32
GSM615316	1	1	0	1	2	3	67
GSM615317	1	1	0	1	3	3	47
GSM615318	1	1	0	1	2	3	47
GSM615319	1	1	0	1	3	3	63

GSM615320	1	0	0	1	3	1	40
GSM615321	1	0	0	1	3	1	50
GSM615322	1	1	0	1	3	3	52
GSM615323	1	1	0	1	3	2	32
GSM615326	1	1	0	0	3	3	57
GSM615327	1	0	0	0	3	1	65
GSM615328	1	1	0	1	2	3	45
GSM615329	1	1	0	0	2	2	41
GSM615330	1	1	0	1	3	3	65
GSM615332	1	1	0	1	2	3	66
GSM615333	1	0	0	0	2	1	48
GSM615335	1	0	0	1	3	1	51
GSM615337	1	1	0	1	2	3	46
GSM615339	1	1	0	0	2	3	58
GSM615340	1	1	0	1	3	2	50
GSM615341	1	1	0	1	2	2	47
GSM615342	1	0	0	1	2	1	43
GSM615343	1	1	0	1	3	3	30
GSM615344	1	1	0	1	3	3	58
GSM615345	1	1	0	1	2	3	64
GSM615346	1	0	0	1	3	1	42
GSM615347	1	1	0	0	3	2	72
GSM615348	1	1	0	0	3	3	33
GSM615349	1	1	0	0	2	2	50
GSM615350	1	1	0	0	3	2	59
GSM615351	1	1	0	0	1	2	53
GSM615352	1	1	0	1	1	2	73
GSM615355	1	1	0	0	2	2	47
GSM615356	1	1	0	0	3	3	43
GSM615358	1	1	0	0	2	3	69
GSM615360	1	1	0	1	2	3	53
GSM615361	1	1	0	1	3	3	64
GSM615362	1	1	0	0	1	2	52
GSM615363	1	0	0	0	3	1	36
GSM615364	1	0	0	1	3	1	34
GSM615365	1	1	0	1	2	2	63
GSM615366	1	1	0	1	1	3	46
GSM615367	1	1	0	1	3	2	49
GSM615368	1	0	0	1	3	1	28
GSM615369	1	0	0	1	3	1	50
GSM615370	1	1	0	0	2	2	36
GSM615372	1	0	0	1	3	1	39
GSM615373	1	1	0	1	2	2	44
GSM615374	1	1	0	0	1	3	56
GSM615376	1	0	0	1	2	1	51

GSM615377	1	1	0	0	2	3	49
GSM615378	1	1	0	1	3	3	41
GSM615380	1	1	0	1	3	3	40
GSM615381	1	1	0	1	3	3	62
GSM615382	1	0	0	0	3	1	59
GSM615384	1	0	0	1	3	1	57
GSM615385	1	0	0	1	2	1	37
GSM615387	1	1	0	1	3	3	38
GSM615388	1	1	0	0	2	3	40
GSM615389	1	1	0	1	1	2	53
GSM615390	1	0	0	1	3	1	46
GSM615391	1	1	0	0	3	3	58
GSM615392	1	0	0	1	3	1	65
GSM615395	1	1	0	0	2	2	64
GSM615398	1	1	0	1	3	3	49
GSM615399	1	1	0	1	2	3	51
GSM615400	1	1	0	1	2	2	73
GSM615401	1	1	0	1	3	2	71
GSM615402	1	1	0	1	2	2	47
GSM615403	1	0	0	1	2	1	50
GSM615404	1	1	0	1	3	2	65
GSM615405	1	1	0	1	3	2	58
GSM615631	1	1	0	0	2	3	32.2
GSM615638	1	1	0	1	3	3	57.5
GSM615640	1	0	0	0	3	1	49
GSM615641	1	0	0	1	3	1	40.3
GSM615642	1	1	0	0	3	2	54
GSM615648	1	0	0	0	3	1	59.2
GSM615657	1	1	0	1	3	3	43.9
GSM615664	1	1	0	1	3	3	44.4
GSM615667	1	1	0	1	3	3	55.7
GSM615674	1	0	0	1	3	1	32.6
GSM615685	1	1	0	1	3	2	62
GSM615686	1	1	0	0	2	2	37
GSM615687	1	0	0	0	3	1	39
GSM615688	1	1	0	0	2	2	38
GSM615689	1	0	0	0	3	1	40
GSM615690	1	1	0	0	2	2	45
GSM615691	1	0	0	1	3	1	34
GSM615692	1	1	0	1	2	2	59
GSM615693	1	1	0	1	3	2	36
GSM615696	1	0	0	1	3	1	65
GSM615697	1	1	0	1	3	2	52
GSM615698	1	1	0	0	3	2	56
GSM615699	1	0	0	1	3	1	47

GSM615700	1	1	0	0	1	2	69
GSM615701	1	0	0	1	3	1	53
GSM615702	1	1	0	0	3	2	57
GSM615703	1	1	0	0	3	2	58
GSM615704	1	1	0	1	1	2	58
GSM615705	1	1	0	1	1	3	40
GSM615707	1	1	0	0	3	2	63
GSM615708	1	1	0	1	2	3	64
GSM615709	1	1	0	1	3	3	27
GSM615710	1	1	0	0	2	2	42
GSM615711	1	1	0	0	2	3	34
GSM615712	1	0	0	1	3	1	57
GSM615713	1	1	0	1	2	3	45
GSM615714	1	0	0	1	3	1	54
GSM615715	1	0	0	0	3	1	50
GSM615716	1	0	0	1	3	1	33
GSM615717	1	1	0	1	3	3	45
GSM615718	1	1	0	1	2	2	54
GSM615719	1	1	0	0	1	2	49
GSM615720	1	1	0	1	3	3	35
GSM615721	1	1	0	0	2	3	60
GSM615722	1	1	0	1	2	3	43
GSM615725	1	1	0	0	3	2	33
GSM615726	1	1	0	1	3	2	58
GSM615728	1	1	0	1	3	3	45
GSM615729	1	1	0	1	3	3	53
GSM615730	1	0	0	0	3	1	47
GSM615731	1	1	0	0	2	2	43
GSM615732	1	0	0	1	3	1	24
GSM615734	1	1	0	0	2	2	61
GSM615735	1	1	0	1	3	3	51
GSM615738	1	1	0	0	2	2	61
GSM615739	1	0	0	0	3	1	53
GSM615740	1	1	0	0	2	3	42
GSM615741	1	1	0	0	3	3	44
GSM615743	1	1	0	1	2	3	43.4
GSM615745	1	1	0	0	2	3	46
GSM615746	1	0	0	1	2	1	56
GSM615747	1	1	0	0	2	3	42
GSM615749	1	1	0	1	3	2	47
GSM615750	1	1	0	1	3	2	27
GSM615751	1	1	0	1	3	3	38
GSM615752	1	1	0	0	2	3	64
GSM615753	1	1	0	1	3	3	46
GSM615754	1	1	0	1	3	3	59

GSM615755	1	1	0	1	3	3	31
GSM615771	1	1	0	1	3	3	39
GSM615772	1	1	0	1	3	3	53
GSM615774	1	1	1	1	2	3	59
GSM615776	1	0	0	1	3	1	45
GSM615777	1	1	0	1	2	2	35
GSM615778	1	1	0	0	3	3	38
GSM615781	1	1	0	0	2	3	63
GSM615783	1	1	0	0	2	2	47
GSM615784	1	1	0	1	3	3	63
GSM615785	1	0	1	1	2	4	51
GSM615786	1	0	0	1	3	1	55
GSM615787	1	0	0	1	3	1	46
GSM615788	1	1	0	1	3	3	71
GSM615789	1	1	0	1	2	2	38
GSM615791	1	1	0	1		2	46
GSM615792	1	1	0	0		2	36
GSM615794	1	1	0	1	2	3	66
GSM615795	1	1	0	1	2	3	39
GSM615796	1	1	1	1	2	3	48
GSM615797	1	1	0	1	2	3	52
GSM615799	1	1	0	1		3	42
GSM615800	1	1	0	0	3	3	40
GSM615801	1	0	0	1	2	1	59
GSM615804	1	0	0	1	3	1	35
GSM615805	1	0	1	1		4	63
GSM615806	1	1	0	1		3	44
GSM615807	1	1	0	1	1	2	41
GSM615808	1	1	0	1	3	2	69
GSM615809	1	1	0	1	2	3	53
GSM615810	1	1	0	1	3	2	37
GSM615811	1	1	0	1	3	2	61
GSM615812	1	0	0	1		1	51
GSM615813	1	0	0	0	3	1	68
GSM615814	1	1	0	0	2	2	67
GSM615815	1	1	0	0	2	3	37
GSM615816	1	1	0	0	1	2	60
GSM615817	1	1	0	0	1	2	42
GSM615818	1	1	0	0	2	3	61
GSM615819	1	1	0	1	2	3	39
GSM615820	1	1	0	1	2	3	42
GSM615821	1	1	0	1	3	3	64
GSM615823	1	1	0	1	3	3	45
GSM615825	1	1	0	1	3	2	42
GSM615826	1	1	0	1	1	3	44

GSM615827	1	1	0	1	3	3	71
GSM615828	1	1	0	0	1	3	45
GSM549233	1	0	0	0	3	1	52
GSM549234	1	0	0	0	3	1	50
GSM549235	1	1	0	1	3	2	50
GSM549239	1	1	0	0	3	3	55
GSM549241	1	1	0	1	2	2	43
GSM549243	1	1	0	0	2	3	43
GSM549244	1	1	0	0	3	3	45
GSM549245	1	0	0	1	3	1	32
GSM549246	1	0	0	1	2	1	38
GSM549247	1	1	0	0	2	3	34
GSM549248	1	0	0	1	2	1	55
GSM549249	1	1	0	1	3	3	60
GSM549250	1	0	0	0	3	1	38
GSM549260	1	0	0	1	3	1	45
GSM549261	1	1	0	1	2	3	39
GSM549262	1	0	0	0	2	1	67
GSM549265	1	0	0		3	1	55
GSM549266	1	1	0		3	3	51
GSM549268	1	1	0		1	3	51
GSM549269	1	1	0		1	2	49
GSM549270	1	1	0		2	3	68
GSM549271	1	0	0		3	1	45
GSM549272	1	1	0			3	73
GSM549273	1	1	0			3	46
GSM549274	1	1	0		3	2	78
GSM549283	1	1	0		2	3	65
GSM549294	1	1	0		2	3	53
GSM549295	1	1	0		2	3	34
GSM750735	1	0	1	1	3	4	66
GSM750736	1	0	1	0	3	4	63
GSM750739	1	0	1	1	3	4	38
GSM750741	1	1	1	1	2	3	63
GSM750742	1	0	1	0	3	4	55
GSM750743	1	0	1	1	2	4	47
GSM750744	1	0	1	1	3	4	46
GSM750748	1	1	1		2	3	
GSM750749	1	1	1		3	3	30
GSM1232992	1	1	1		3	3	66
GSM1232993	1	0	1		2	4	58
GSM1233005	1	1	1		3	3	57
GSM1233007	1	0	1		3	4	54
GSM1233010	1	1	0		2	2	69
GSM1233013	1	0	1		3	4	68

GSM1233018	1	0	1		2	4	55
GSM1233019	1	0	1		3	4	56
GSM1233021	1	1	1		3	3	66
GSM1233025	1	1	0		2	2	45
GSM1233029	1	0	1		2	4	67
GSM1233030	1	0	0		2	1	59
GSM1233031	1	0	1		3	4	63
GSM1233032	1	0	0		2	1	46
GSM1233035	1	1	0		3	2	32
GSM1233038	1	0	0		3	1	46
GSM1233039	1	0	1		2	4	49
GSM1233042	1	1	1		2	3	36
GSM1233043	1	1	1		2	3	51
GSM1233044	1	1	0		2	2	55
GSM1233046	1	0	0		3	1	53
GSM1233051	1	1	1		3	3	51
GSM1233054	1	1	1		3	3	48
GSM1233057	1	0	1		2	4	33
GSM1233060	1	0	1		2	4	58
GSM1233062	1	0	1		3	4	49
GSM1233067	1	1	0		3	2	71
GSM1233069	1	1	0		2	2	46
GSM1233072	1	1	0		2	2	60
GSM1233075	1	1	1		2	3	46
GSM1233078	1	1	0		2	3	35
GSM1233079	1	1	0		3	3	41
GSM1233082	1	0	1		2	4	62
GSM1233083	1	1	0		3	2	51
GSM1233086	1	1	0		3	2	62
GSM1233091	1	0	1		3	4	54
GSM1233095	1	0	1		3	4	55
GSM1233096	1	0	1		3	4	38
GSM1233101	1	1	1		2	3	55
GSM1233102	1	0	0		3	1	49
GSM1233103	1	0	0		3	1	60
GSM1233105	1	1	0		3	2	71
GSM1233107	1	0	0		3	1	46
GSM1233108	1	1	0		3	3	54
GSM1233109	1	1	0		2	2	49
GSM1233110	1	1	0		2	2	47
GSM1233113	1	1	0		3	3	53
GSM1233116	1	1	0		2	2	34
GSM1233117	1	1	0		3	2	54
GSM1233118	1	1	0		2	2	46
GSM1233120	1	0	0		3	1	38

GSM1233121	1	1	0		2	2	70
GSM1233123	1	1	0		2	2	71
GSM1233124	1	1	0		3	3	45
GSM1233125	1	1	0		2	2	46
GSM1233126	1	0	0		3	1	64
GSM1233127	1	0	0		3	1	42
GSM1233128	1	1	0		3	3	44
GSM1233130	1	1	0		2	2	47
GSM1233131	1	0	1		3	4	57
GSM1233133	1	1	0		2	2	42
GSM1233134	1	1	0		3	2	64
GSM1233135	1	1	0		2	2	66
GSM1233136	1	1	0		2	2	56
GSM1233137	1	1	0		2	2	60
GSM1233138	1	0	0		3	1	56
GSM1233140	1	1	0		2	2	51
GSM1233141	1	0	0		3	1	49
GSM1233142	1	0	0		3	1	38
GSM1233144	1	1	0		3	3	47
GSM1233147	1	1	0			2	42
GSM1232994	1	0	1		2	4	55
GSM1232996	1	0	1		2	4	73
GSM1232997	1	1	1		3	3	49
GSM1232998	1	0	0		3	1	47
GSM1232999	1	1	1		3	3	60
GSM1233000	1	0	1		3	4	53
GSM1233004	1	1	0		3	2	67
GSM1233011	1	1	1		2	3	59
GSM1233012	1	1	0		2	2	47
GSM1233023	1	1	1		3	3	51
GSM1233027	1	0	0		2	1	52
GSM1233033	1	0	0		3	1	48
GSM1233036	1	1	0		3	3	44
GSM1233037	1	0	1		3	4	39
GSM1233041	1	0	0		3	1	46
GSM1233045	1	0	1		2	4	35
GSM1233047	1	1	1		2	3	35
GSM1233050	1	0	1		3	4	50
GSM1233052	1	0	1		2	4	41
GSM1233053	1	1	0		2	3	54
GSM1233055	1	1	1		2	3	52
GSM1233061	1	1	1		2	3	45
GSM1233063	1	0	0		3	1	37
GSM1233065	1	1	1		2	3	42
GSM1233070	1	0	1		2	4	55

GSM1233077	1	1	0		2	2	58
GSM1233081	1	1	0		3	3	46
GSM1233084	1	0	0		3	1	36
GSM1233087	1	1	1		3	3	52
GSM1233089	1	1	0		2	2	49
GSM1233099	1	0	0		3	1	52
GSM1233112	1	1	0		2	2	58
GSM411393	1	1	1	1	2	3	
GSM411394	1	0	0	0	3	1	
GSM411395	1	0	1	1	2	4	
GSM411396	1	0	0	1	3	1	
GSM411397	1	0	0	1	3	1	
GSM411398	1	0	0	0	3	1	
GSM411400	1	1	0	1	3	2	
GSM411401	1	0	0	0	3	1	
GSM411402	1	0	0	0	2	1	
GSM411403	1	0	0	1	3	1	
GSM411404	1	0	1	1	3	4	
GSM411405	1	0	1	1		4	
GSM411406	1	1	1	1	3	3	
GSM411407	1	0	1	0	3	4	
GSM411408	1	0	0	0	3	1	
GSM411409	1	0	0	1	3	1	
GSM505387	1	0	1	0	3	4	55
GSM505391	1	1	1	0	2	3	56
GSM505437	1	0	1	1	3	4	51
GSM505438	1	0	0	1	3	1	38
GSM505439	1	0	0	1	2	1	71
GSM505445	1	0	1	1	3	4	45
GSM505496	1	1	0			3	64
GSM508012	1	0	0	1	3	1	48
GSM508036	1	1	0	1		3	43
GSM508038	1	1	0	1		3	46
GSM508071	1	1	0	1	3	3	59
GSM508087	1	0	0	0	3	1	59
GSM508099	1	0	1	0	3	4	49
GSM508150	1	1	0	0		3	45
GSM508151	1	0	1	0		4	64
GSM508152	1	0	0	0	3	1	49
GSM508164	1	1	1	1	3	3	48
GSM508165	1	0	0	0	3	1	45
GSM508170	1	1	0	1	2	3	53
GSM809185	1	0	0	1	3	1	56
GSM809192	1	0	1	0	2	4	55
GSM809194	1	1	0	1	2	3	36

GSM809202	1	0	1	1	2	4	65
GSM809203	1	0	0	1	2	1	52
GSM809213	1	1	0	1	1	2	46
GSM809225	1	0	0	1	3	1	61
GSM809226	1	0	1	1	2	4	62
GSM809229	1	1	0	1	2	3	53
GSM809231	1	0	0	1	2	1	28
GSM809232	1	0	0	1	2	1	68
GSM809236	1	0	0	1	2	1	47
GSM809237	1	1	0	1	3	3	63
GSM809238	1	0	1	1	2	4	56
GSM809246	1	0	1	0	2	4	50
GSM809248	1	1	0	1	2	2	36
GSM809253	1	1	0	0	1	3	54
GSM809256	1	0	1	1	3	4	50
GSM809266	1	0	0	1	2	1	70
GSM809271	1	1	1	1	2	3	61
GSM809272	1	0	1	1	2	4	49
GSM809274	1	0	0	1	3	1	53
GSM809280	1	0	0	1	3	1	60
GSM809289	1	1	1	0	3	3	48
GSM809294	1	0	1	1	2	4	61
GSM809295	1	0	0	1	3	1	68
GSM809298	1	1	0	1	2	2	61
GSM465183	1	1	0			2	
GSM465189	1	1	0			2	
GSM465199	1	1	0			2	
GSM465204	1	1	0			2	
GSM465213	1	1	0			2	
GSM465224	1	1	0			2	
GSM465231	1	1	0			2	
GSM465236	1	1	0			2	
GSM465242	1	0	0			1	
GSM465185	1	1	0			2	
GSM465188	1	1	0			2	
GSM465214	1	1	0			2	
GSM465222	1	1	0			2	
GSM465225	1	1	0			2	
GSM465243	1	0	0			1	
GSM590860	1	0	0	1	3	1	51.132
GSM1030085	1	0	0			1	34
GSM1030086	1	0	0			1	50
GSM1030087	1	0	0			1	45
GSM1030088	1	0	1			4	49
GSM1030089	1	0	0			1	52

GSM1030090	1	0	0			1	31
GSM1030091	1	0	1			4	52
GSM1030092	1	0	1			4	56
GSM1030094	1	0	1			4	27
GSM1030095	1	1	1			3	56
GSM1030096	1	0	1			4	65
GSM1030097	1	0	1			4	61
GSM1030099	1	1	0			2	67
GSM1030100	1	1	0			2	45
GSM1030101	1	1	0			2	48
GSM1030102	1	1	1			3	46
GSM1030103	1	1	0			2	41
GSM1030104	1	0	1			4	60
GSM1030105	1	1	0			2	47
GSM1030106	1	1	0			2	65
GSM1030109	1	1	0			2	45
GSM1030110	1	1	0			2	48
GSM1030113	1	1	0			2	42
GSM1030114	1	1	0			2	46
GSM1030115	1	1	0			2	56
GSM1030116	1	1	0			2	59
GSM1030118	1	1	0			2	44
GSM1030119	1	0	1			4	47
GSM1030121	1	1	0			2	48
GSM1030122	1	1	0			2	54
GSM1030123	1	1	0			2	55
GSM1030126	1	1	1			3	56
GSM1030128	1	1	0			2	47
GSM1030131	1	1	0			2	68
GSM1030132	1	1	0			2	68
GSM1030136	1	1	0			2	67
GSM1030138	1	1	0			2	54
GSM1030139	1	1	0			2	79
GSM1030140	1	1	0			2	47
GSM1030141	1	1	0			3	46
GSM1030144	1	1	0			2	48
GSM1030145	1	1	0			2	44
GSM1030146	1	1	0			2	56
GSM1030147	1	1	0			2	63
GSM1030149	1	1	0			2	68
GSM1030150	1	1	0			2	47
GSM1030151	1	1	0			2	70
GSM1030152	1	1	0			2	48
GSM1030153	1	1	0			2	56
GSM1030155	1	1	0			2	62

GSM1030156	1	0	0			1	31
GSM1030157	1	0	0			1	59
GSM1030158	1	0	0			1	40
GSM1030159	1	1	0			2	62
GSM1030160	1	0	0			1	45
GSM1030162	1	0	0			1	54
GSM1030163	1	0	0			1	50
GSM1030164	1	0	0			1	44
GSM1030166	1	0	0			1	54
GSM1030167	1	0	0			1	39
GSM1030168	1	0	0			1	43
GSM1030169	1	0	0			1	47
GSM1030170	1	0	0			1	65
GSM1030171	1	0	0			1	49
GSM1030172	1	0	0			1	32
GSM1030173	1	0	0			1	46
GSM1030174	1	0	0			1	35
GSM1030175	1	0	0			1	48
GSM1030176	1	0	0			1	61
GSM1030177	1	0	0			1	32
GSM1030179	1	1	0			2	49
GSM1030180	1	0	0			1	36
GSM1030182	1	1	0			3	38
GSM1030183	1	0	0			1	40
GSM1030185	1	0	0			1	56
GSM1030186	1	1	0			2	47
GSM1030187	1	0	0			1	29
GSM1030188	1	1	0			2	35
GSM1030189	1	0	0			1	59
GSM1030190	1	0	0			1	43
GSM1030191	1	0	0			1	43
GSM1030195	1	0	0			1	63
GSM1030196	1	0	0			1	42
GSM1030200	1	0	0			1	45
GSM1030201	1	1	0			2	43
GSM1030202	1	1	0			2	63
GSM1030203	1	0	0			1	35
GSM1030204	1	0	0			1	49
GSM1030205	1	0	0			1	31
GSM1030206	1	0	0			1	60
GSM1030207	1	0	0			1	66
GSM1030209	1	1	0			3	36
GSM1030210	1	0	0			1	62
GSM1030212	1	0	0			1	40
GSM1030213	1	0	0			1	55

GSM1030215	1	0	0			1	25
GSM1030217	1	1	1			3	43
GSM1030218	1	1	0			2	72
GSM1030219	1	1	0			2	38
GSM1030221	1	1	0			2	37
GSM1030222	1	1	0			2	56
GSM1030223	1	0	0			1	44
GSM1030224	1	1	0			2	36
GSM1030225	1	0	0			1	43
GSM1030226	1	1	0			2	35
GSM1030228	1	0	0			1	28
GSM1030229	1	0	0			1	40
GSM1030230	1	1	0			2	56
GSM1030233	1	1	0			2	57
GSM1030234	1	0	0			1	67
GSM1030235	1	0	1			4	73
GSM1030236	1	0	1			4	33
GSM1030238	1	0	1			4	55
GSM1030240	1	1	0			2	63
GSM1030241	1	1	0			2	33
GSM1030242	1	1	0			2	33
GSM1030243	1	1	0			2	33
GSM1030247	1	1	0			2	41
GSM1030248	1	0	1			4	55
GSM1030249	1	1	0			2	44
GSM1030250	1	1	0			2	54
GSM1030251	1	1	0			2	39
GSM1030253	1	1	0			2	41
GSM1030257	1	1	0			2	45
GSM1030260	1	1	0			3	50
GSM1030261	1	1	0			2	58
GSM1030262	1	1	0			2	39
GSM1030263	1	1	0			3	56
GSM1030264	1	1	0			2	48
GSM1030265	1	1	0			2	46
GSM1030266	1	1	0			2	43
GSM1030267	1	1	0			2	42
GSM1030270	1	1	0			2	58
GSM1030271	1	1	0			2	59
GSM1030272	1	1	0			2	57
GSM1030273	1	1	0			2	38
GSM1030274	1	1	0			2	43
GSM1030275	1	1	0			2	58
GSM1030276	1	1	0			3	34
GSM1030277	1	1	0			2	65

GSM1030278	1	1	0			2	50
GSM1030279	1	1	0			2	40
GSM1030280	1	1	0			2	70
GSM1030281	1	1	0			2	50
GSM1030282	1	1	0			2	60
GSM1030287	1	1	0			2	54
GSM1030289	1	1	0			2	40
GSM1030290	1	0	0			1	35
GSM1030291	1	1	0			2	47
GSM1030292	1	0	0			1	50
GSM1030293	1	0	0			1	33
GSM1030294	1	0	0			1	54
GSM1030296	1	0	0			1	42
GSM1030298	1	0	0			1	53
GSM1030299	1	0	0			1	32
GSM1030301	1	0	0			1	48
GSM1030303	1	0	0			1	37
GSM1030304	1	0	0			1	46
GSM1030305	1	0	0			1	44
GSM1030306	1	0	0			1	52
GSM1030310	1	1	0			2	34
GSM1030311	1	0	0			1	54
GSM1030314	1	0	0			1	65
GSM1030315	1	1	0			2	41
GSM1030316	1	1	0			3	30
GSM1030317	1	0	0			1	44
GSM1030318	1	0	0			1	52
GSM1030319	1	0	0			1	54
GSM1030321	1	1	0			2	57
GSM1030322	1	0	0			1	42
GSM1030323	1	0	0			1	38
GSM1030324	1	0	0			1	50
GSM1030327	1	0	0			1	37
GSM1030329	1	1	0			3	41
GSM1030330	1	0	0			1	60
GSM1030331	1	0	0			1	51
GSM1030332	1	1	0			2	40
GSM1030333	1	0	0			1	48
GSM1030334	1	0	0			1	45
GSM1030337	1	0	0			1	41
GSM1030339	1	0	0			1	45
GSM1030340	1	0	0			1	50
GSM1030341	1	0	0			1	32
GSM1030343	1	0	0			1	48
GSM1030344	1	0	0			1	46

GSM1030345	1	1	0			3	46
GSM1030348	1	0	0			1	53
GSM1030349	1	0	0			1	73
GSM1030350	1	0	1			4	46
GSM1030351	1	0	0			1	61
GSM1030352	1	0	1			4	35
GSM1030353	1	0	0			1	56
GSM1030355	1	1	0			2	44
GSM1030356	1	1	0			2	44
GSM1030357	1	1	0			2	41
GSM505349	1	0	1		3	4	49
GSM505463	1	1	1		2	3	70
GSM505342	1	0	1		3	4	59
GSM505341	1	1	1		3	3	44
GSM505373	1	1	1		3	3	54
GSM505335	1	1	1		3	3	42
GSM505507	1	1	0		3	2	49
GSM505390	1	1	1		2	3	56
GSM505386	1	0	1		3	4	55
GSM505511	1	0	1		2	4	53
GSM505419	1	0	1		3	4	62
GSM505434	1	1	1		3	3	45
GSM505435	1	0	0		2	1	71
GSM505459	1	1	0		3	2	62
GSM505447	1	0	1	0	3	4	51
GSM505521	1	0	1		3	4	55
GSM505531	1	1	1		3	3	46
GSM505436	1	1	0		3	3	48
X1719d_U133A	1	0	0		3	1	31
X2946_U133A	1	0	1		3	4	48
X6860_U133A	1	1	1		3	3	40
X6868_U133A	1	0	1		3	4	65
X6871_U133A	1	0	0		3	1	46
X3886_U133A	1	0	0		3	1	63
X5726_U133A	1	0	1		3	4	47
X5687_U133A	1	0	0		3	1	50
X4711_U133A	1	1	1		3	3	61
X4204_U133A	1	0	0		3	1	52
X2148_U133A	1	0	1		3	4	55
GSM930525	1	0	0	0	3	1	50
GSM930526	1	1	1	0	3	3	34
GSM930527	1	1	1	0	3	3	52
GSM930528	1	0	1	0	3	4	30
GSM930529	1	1	1	1	3	3	38
GSM930530	1	1	1	0	3	3	36

GSM930531	1	1	1	0	2	3	47
GSM930532	1	0	1	0	3	4	56
GSM930533	1	0	1	0	3	4	45
GSM930535	1	1	1	0	3	3	43
GSM930536	1	1	1	0	2	3	38
GSM930537	1	1	1	0	2	3	63
GSM930538	1	0	0	0	3	1	50
GSM930539	1	1	1	0	2	3	41
GSM930540	1	1	1	0	2	3	53
GSM930541	1	1	1	0	3	3	51
GSM930542	1	0	1	0	3	4	55
GSM930543	1	0	1	0	3	4	62
GSM930544	1	0	1	0	3	4	53
GSM930545	1	1	0	0	2	2	56
GSM930546	1	0	1	0		4	46
GSM930547	1	1	1	0	3	3	56
GSM930548	1	1	1	0	2	3	52
GSM615099	1	0	0	0		1	40.8
GSM615110	1	1	0	0	3	3	49.6
GSM615122	1	1	0	0	2	3	31.3
GSM615123	1	1	0	0	3	3	53.1
GSM615137	1	1	0	0		3	35.1
GSM615139	1	0	0	0	3	1	56.3
GSM615140	1	1	0	0	2	3	39.4
GSM615143	1	0	0	0	3	1	42.7
GSM615146	1	1	0		3	3	38.9
GSM615154	1	0	0	0	3	1	40.8
GSM615164	1	0	0	0	3	1	42.1
GSM615167	1	1	0	0	2	3	34.4
GSM615171	1	0	0	0		1	43.1
GSM615175	1	1	0	0	3	3	42.5
GSM615181	1	0	0	1	3	1	49
GSM615186	1	1	0	1	1	3	51
GSM615191	1	0	0	0	3	1	37
GSM615193	1	1	0	0	3	2	40
GSM615194	1	0	0	1	3	1	61
GSM615201	1	1	0	0	3	3	41
GSM615202	1	1	0	1	3	3	42
GSM615209	1	0	1	1	3	4	56
GSM615211	1	0	0	1	3	1	35
GSM615212	1	0	0	0	3	1	48
GSM615213	1	0	0	1	3	1	53
GSM615215	1	0	0	1	3	1	44
GSM615240	1	0	0	1	3	1	48
GSM615242	1	0	0	1	3	1	46

GSM615256	1	1	0	1	3	2	51
GSM615263	1	0	0	1	3	1	61
GSM615264	1	0	0	0	3	1	45
GSM615270	1	0	0	1	3	1	38
GSM615280	1	1	0	1	3	2	41
GSM615284	1	0	0	1	3	1	50
GSM615291	1	0	0	1	3	1	59
GSM615293	1	0	0	1	3	1	31
GSM615300	1	0	0	1	3	1	57
GSM615307	1	0	0	1	3	1	32
GSM615310	1	0	0	1	3	1	64
GSM615324	1	0	0	1	3	1	49
GSM615325	1	0	0	1	3	1	43
GSM615331	1	1	0	1	3	3	61
GSM615334	1	0	0	1	3	1	61
GSM615336	1	0	0	1	3	1	49
GSM615338	1	0	0	1	3	1	57
GSM615353	1	1	0	1	3	3	46
GSM615354	1	0	0	1	2	1	46
GSM615357	1	0	0	1	3	1	53
GSM615359	1	0	0	1	3	1	51
GSM615371	1	0	0	1	3	1	67
GSM615375	1	1	0	1	3	3	75
GSM615379	1	0	0	1	3	1	62
GSM615383	1	1	0	0	2	2	51
GSM615393	1	0	0	1	3	1	43
GSM615394	1	0	0	0	3	1	62
GSM615396	1	0	0	1	3	1	32
GSM615397	1	0	0	1	2	1	57
GSM615635	1	1	0	1	3	3	43.8
GSM615658	1	0	0	1	3	1	50.3
GSM615660	1	0	0	1	3	1	40.7
GSM615665	1	1	0	1	2	3	50.1
GSM615672	1	0	0	0		1	49.6
GSM615694	1	0	0	1	3	1	40
GSM615695	1	0	0	1	3	1	72
GSM615706	1	0	0	1	2	1	43
GSM615723	1	1	0	1	3	3	41
GSM615724	1	1	0	0	3	3	59
GSM615727	1	1	0	1	3	3	24
GSM615733	1	0	0	0	3	1	64
GSM615736	1	1	0	1	3	3	52
GSM615737	1	1	0	0	3	3	50
GSM615742	1	0	0	1	3	1	38
GSM615744	1	1	0	0	3	3	48

GSM615748	1	1	0	0	3	3	60
GSM615773	1	1	0	1	2	3	58
GSM615775	1	1	1	0	3	3	46
GSM615779	1	1	0	0	2	3	56
GSM615780	1	1	0	1	3	2	39
GSM615782	1	1	0	1	3	3	59
GSM615793	1	0	0	0		1	53
GSM615798	1	0	0	1	3	1	40
GSM615802	1	1	0	1		3	44
GSM615803	1	0	1	1		4	58
GSM615822	1	1	0	1		2	56
GSM615824	1	1	0	0	3	2	46
GSM549230	1	0	0		3	1	55
GSM549231	1	1	0		3	3	52
GSM549232	1	1	0		3	3	62
GSM549236	1	1	0		3	3	45
GSM549237	1	0	1		3	4	75
GSM549238	1	0	0	0	3	1	50
GSM549240	1	1	0	0	2	3	31
GSM549242	1	0	0	0	3	1	57
GSM549251	1	0	0	0	3	1	37
GSM549252	1	0	0		3	1	32
GSM549253	1	0	0		3	1	55
GSM549254	1	0	0	0	3	1	38
GSM549255	1	1	0	0	2	3	43
GSM549256	1	0	0	1	3	1	47
GSM549257	1	0	0	0	3	1	54
GSM549258	1	1	0	1	3	3	47
GSM549259	1	1	0	1	2	3	46
GSM549263	1	1	0		3	2	53
GSM549264	1	1	0		3	2	66
GSM549267	1	0	0		3	1	33
GSM549286	1	1	0		2	2	54
GSM750734	1	1	1		3	3	55
GSM750738	1	1	1	0	3	3	51
GSM750745	1	1	1	1	3	3	38
GSM750746	1	1	1	1	2	3	54
GSM1232995	1	1	1		2	3	49
GSM1233002	1	0	1		3	4	52
GSM1233003	1	1	0		3	3	58
GSM1233014	1	1	0		3	2	71
GSM1233015	1	0	1		3	4	63
GSM1233016	1	1	0		2	3	52
GSM1233024	1	1	0		2	2	46
GSM1233049	1	1	1		3	3	48

GSM1233064	1	1	1		3	3	56
GSM1233068	1	0	1		3	4	80
GSM1233073	1	0	1		3	4	55
GSM1233085	1	1	0		2	3	39
GSM1233093	1	0	1		3	4	56
GSM1233098	1	1	1		3	3	69
GSM1233114	1	0	0		3	1	30
GSM1233115	1	0	1		3	4	35
GSM1233119	1	0	0		3	1	44
GSM1233129	1	1	1		2	3	55
GSM1233132	1	0	0		3	1	52
GSM1233139	1	0	0		3	1	55
GSM1233143	1	1	0		3	2	48
GSM1233145	1	0	0		3	1	46
GSM1233001	1	0	1		3	4	44
GSM1233006	1	1	1		3	3	51
GSM1233008	1	0	1		2	4	64
GSM1233009	1	0	1			4	50
GSM1233017	1	0	1		3	4	60
GSM1233020	1	1	1		2	3	59
GSM1233022	1	0	1		3	4	54
GSM1233026	1	0	0		2	1	48
GSM1233028	1	0	1		2	4	64
GSM1233034	1	0	1		3	4	53
GSM1233040	1	1	1		3	3	48
GSM1233048	1	0	1		2	4	47
GSM1233056	1	1	0		2	2	56
GSM1233058	1	0	0		3	1	45
GSM1233059	1	0	1		2	4	57
GSM1233066	1	1	0		2	2	35
GSM1233071	1	0	1		3	4	41
GSM1233074	1	1	1		3	3	55
GSM1233076	1	1	0		3	3	45
GSM1233080	1	0	1		2	4	58
GSM1233088	1	1	1		2	3	41
GSM1233090	1	1	1		3	3	56
GSM1233092	1	1	1		3	3	56
GSM1233094	1	0	1		3	4	60
GSM1233097	1	0	1		2	4	58
GSM1233100	1	0	1		2	4	46
GSM1233104	1	0	0		2	1	42
GSM1233106	1	0	1		2	4	26
GSM1233111	1	1	0		3	2	76
GSM1233122	1	1	1		3	3	38
GSM1233146	1	1	1			3	58

Supplementary Table 2. Association of other relevant immune-related genes and chemotherapy response on triple negative breast cancer patients.

	ROC plotter		
**geneName**	**AUC**	**p-value**	
**CTLA-4**	**0.674**	6.3e-03	**Responders**
**CD274**	**0.648**	1.7e-02	**Responders**
**PDCD1**	**0.63**	1.4e-03	**Non-responders**
**HAVcr2**	0.507	0.47	
**CD276**	0.59	0.12	
**VTCN1**	0.505	0.46	
**BTLA**	0.59	0.11	
**ADRA2A**	0.574	0.051	
**CD244**	0.51	0.41	
**TIGIT**	**0.658**	1.6e-02	**Responders**
**CD96**	0.535	0.22	
**TNFRSF9**	0.504	0.47	
**TNFRSF4**	0.532	0.24	
**GITR**	0.55	0.25	
**CD40**	0.595	1.7e-02	
**CD28**	0.553	0.12	
**CD27**	0.564	0.079	
**ICOS**	**0.635**	1.0e-03	**Responders**
**STAT4**	0.522	0.31	
**GATA3**	0.576	4.5e-02	
**RORC**	0.586	0.13	
**FOXP3**	0.564	0.2	
